# Cryogenic electron ptychographic single particle analysis with wide bandwidth information transfer

**DOI:** 10.1038/s41467-023-38268-0

**Published:** 2023-05-25

**Authors:** Xudong Pei, Liqi Zhou, Chen Huang, Mark Boyce, Judy S. Kim, Emanuela Liberti, Yiming Hu, Takeo Sasaki, Peter D. Nellist, Peijun Zhang, David I. Stuart, Angus I. Kirkland, Peng Wang

**Affiliations:** 1 National Laboratory of Solid State Microstructures, Jiangsu Key Laboratory of Artificial Functional Materials, College of Engineering and Applied Sciences and Collaborative Innovation Center of Advanced Microstructures, Nanjing University, Nanjing, China; 2 Department of Physics, University of Warwick, Coventry, UK; 3 The Rosalind Franklin Institute, Harwell Science and Innovation Campus, Didcot, UK; 4 Division of Structural Biology, Welcome Trust Centre for Human Genetics, University of Oxford, Oxford, UK; 5 Department of Materials, University of Oxford, Oxford, UK; 6 JEOL Ltd, Tokyo, Japan; 7 Diamond Light Source, Harwell Science and Innovation Campus, Didcot, UK

**Keywords:** Transmission electron microscopy, Cryoelectron microscopy, Transmission electron microscopy

## Abstract

Advances in cryogenic transmission electron microscopy have revolutionised the determination of many macromolecular structures at atomic or near-atomic resolution. This method is based on conventional defocused phase contrast imaging. However, it has limitations of weaker contrast for small biological molecules embedded in vitreous ice, in comparison with cryo-ptychography, which shows increased contrast. Here we report a single-particle analysis based on the use of ptychographic reconstruction data, demonstrating that three dimensional reconstructions with a wide information transfer bandwidth can be recovered by Fourier domain synthesis. Our work suggests future applications in otherwise challenging single particle analyses, including small macromolecules and heterogeneous or flexible particles. In addition structure determination in situ within cells without the requirement for protein purification and expression may be possible.

## Abstract (web summary)

Cryogenic electron ptychography (Cryo-EPty) is an emerging method for imaging the structure of biomacromolecules. Here, authors develop single particle analysis based on cryo-EPty phase for high contrast, wide spatial frequency-band 3D imaging.

## Introduction

Cryo-electron microscopy^1–3^ (cryo-EM), is a powerful method for visualising a wide range of biological macromolecules at near-atomic resolution and is particularly powerful for studying the structure of biologically significant protein complexes which are often difficult to solve using X-ray or nuclear magnetic resonance (NMR) methods^4^. Numerous technical developments including optimized specimen preparation^5,6^, improved image reconstruction and processing algorithms^7–9^, new electron sources and energy filters^10^ and a generation of direct electron detectors have allowed the structures of many biomolecules with weights greater than 100 kDa to be solved at close to atomic resolution^10^, using single particle analysis (SPA)^11^.

However, despite these advances, SPA has been almost exclusively based on the use of phase contrast images recorded at high defocus. A major limitation of this approach is that the low-frequency information from weak-phase objects such as most macromolecules^12^, is attenuated and high frequency information suffers from rapid reversals in the phase contrast transfer function (PCTF). This can cause difficulties in the accurate identification, classification and alignment of particles to a common reference^13^. To overcome the low contrast and signal-to-noise ratio (SNR) in defocused cryo-EM images that has often precluded high resolution 3D reconstructions using the SPA method, it is therefore important to develop high-contrast phase-sensitive imaging modes. Phase plates have been successfully used to increase low-frequency contrast and SNR for in-focus images^13–16^. However, the routine use of phase plates is often restricted by signal attenuation at high frequencies and inconsistent fabrication giving rise to unstable phase shifts^16^.

An alternative strategy is based on the use of phase information recovered using electron ptychography^17^, which has been recently demonstrated under low dose conditions for biological samples embedded in vitreous ice. Ptychography is a lensless imaging approach originally proposed by Hoppe^18^ where a sample is scanned by a suitably conditioned probe in a 2D array. This provides an array of diffraction patterns recorded in the far field as a function of probe position forming a four-dimensional (4D) dataset. Using this 4D dataset, quantitative phase data with high spatial resolution can be recovered commonly using one of several iterative phase retrieval algorithms^19,20^. This method has been used with a variety of radiations including light, X-rays, and electrons, in wide ranging applications in both the physical and life sciences^17,21–24^. Using electrons, ptychography has generated considerable interest given its potential for super-resolution imaging^24,25^, high-contrast light-element detection^26–28^, 3D optical sectioning^28–30^ and potential coupling to spectroscopic data acquisition^31^. Moreover, as ptychography utilizes the full diffraction pattern, it is dose-efficient^17,32,33^ particularly when data is recorded using direct electron detectors^34,35^ which record data with high signal-to-noise at low electron dose. This approach has recently been demonstrated for micrometer wide phase reconstruction of an unstained virus-infected cell at a dose of 27 e/Å^2^ ^17^.

Here we describe a standardised protocol for 3D SPA using ptychographic data recorded under cryogenic conditions (cryo-EPty SPA), and experimentally demonstrate that this can restore 3D information across a wide bandwidth of spatial frequencies by adjusting the convergence semi angle (CSA) of the electron probe. A 3D map with a wide bandwidth in the Fourier domain has been synthesised by combining data for different CSAs that shows better contrast than conventional cryo-EM SPA. We also show, using simulated data that cryo-EPty SPA has future potential for structural studies of small molecules.

## Results

### Protocol for ptychographic SPA

**Fig. 1:**
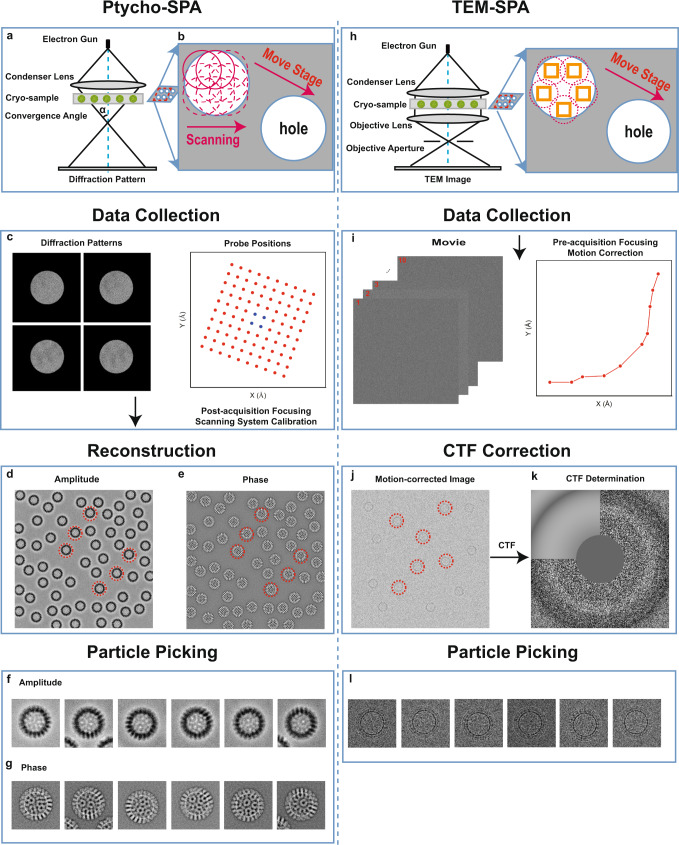
Comparison of workflows for Cryo EPt-SPA (Left column) and TEM-SPA (Right column). Schematic diagrams of the optical configuration **a** and data acquisition for ptychography **b**. **c** Typical diffraction patterns and corresponding probe positions used for ptychography (blue). Ptychographic reconstructed amplitude **d** and phase **e** of an object function. Instances of amplitude **f** and phase **g** of particles picked from **d** and **e**, respectively. **h** Schematic diagram of the optical configuration for TEM data acquisition. **i** Movie frames collected in TEM mode (left) and translations calculated for individual frames (right). **j** Motion-corrected image from a movie. **k** CTF correction by fitting the amplitude spectrum of **j**. **l** Picked particles instances from **j**.

4D data collection: 4D datasets were acquired in a scanning diffraction configuration shown schematically in Fig. 1a, whereby a defocused probe with a known CSA, α was raster scanned over a cryo-sample for a field of view (FOV) that can reach micrometer scale^17^. The defocus value of the probe and the scanning step-size were adjusted such that the overlap ratio between each adjacent illuminated area (Fig. 1b) was maintained in the range between 95%^33^ and 60%^36^ which is essential to ensure computational convergence of the iterative algorithm used^36^. We note that this parameter affects the total dose^32^. A fast direct electron detector^34,35^ that was synchronised with the scanning system of the microscope was used to collect the 4D diffraction datasets (Fig. 1c). Moving from one FOV to the next was achieved by shifting the scanned illumination area, although mechanical movement of the stage could also be used.

Post-acquisition scanning alignment and focusing: The probe positions and defocus values used for data collection are accurately known and considered as prior knowledge in the ptychographic reconstruction used here. However, due to unavoidable instabilities in the microscope^37^ such as scan distortions including jitter, stretching and warping, together with possible mechanical stage drift and defocus drift, the actual values can deviate from their nominal settings as shown in Fig. 1c (right). To overcome this a starting estimate of probe positions can be calculated using cross-correlation^38^ and position refinement algorithms based on conjugate gradient descent methods^39^, annealing algorithms^40^ or serial cross-correlation^41^ can then be included in the iterative reconstruction to eliminate these positional errors.

As the defocus value directly determines the probe size at the sample plane, an accurate initial estimate can speed up the convergence of the reconstruction, even though the probe function can ultimately be retrieved within certain reconstruction algorithms^42^ including that used in this work. The initial defocus value can be accurately estimated from the Ronchigram^43^ or by using a computational multislice algorithm^28–30^ as used here.

Iterative ptychographic reconstruction: Given optimised probe positions and defoci, the object and probe complex functions can be simultaneously retrieved from the 4D datasets using one of several alternative reconstruction methods including iterative methods such as the extended ptychographic iterative engine (ePIE)^20^ or difference map (DM)^22^ or an analytical Wigner distribution deconvolution (WDD)^19^. Here ePIE was used. For thick biological samples, ptychography can also retrieve 3D structural information by computational optical sectioning^28,29^, which has further potential for use in imaging large volumes of biological material. Viral particles have been experimentally reconstructed in 2D using ePIE^17^, demonstrating that both the amplitude and phase of the sample wavefunction can be recovered at low dose for cryogenic samples, as shown in Fig. 1d, e.

Particle picking: From the phase and amplitude of the specimen wavefunction, particle-picking procedures^44–46^ that have been developed for cryo-EM SPA can be directly applied to both. Using both enables a cross-check and can also provide a coordinate reference if one signal is weak. Multiple individual particles can then be sequentially picked from the phase and amplitude and formed into two positionally coordinated stacks of particle phases and amplitudes, as shown in Fig. 1f, g. For the higher CSA data (4.83 mrad), the amplitude was used to aid particle picking in the phase as shown in Supplementary Fig. 1c, f.

**Fig. 2:**
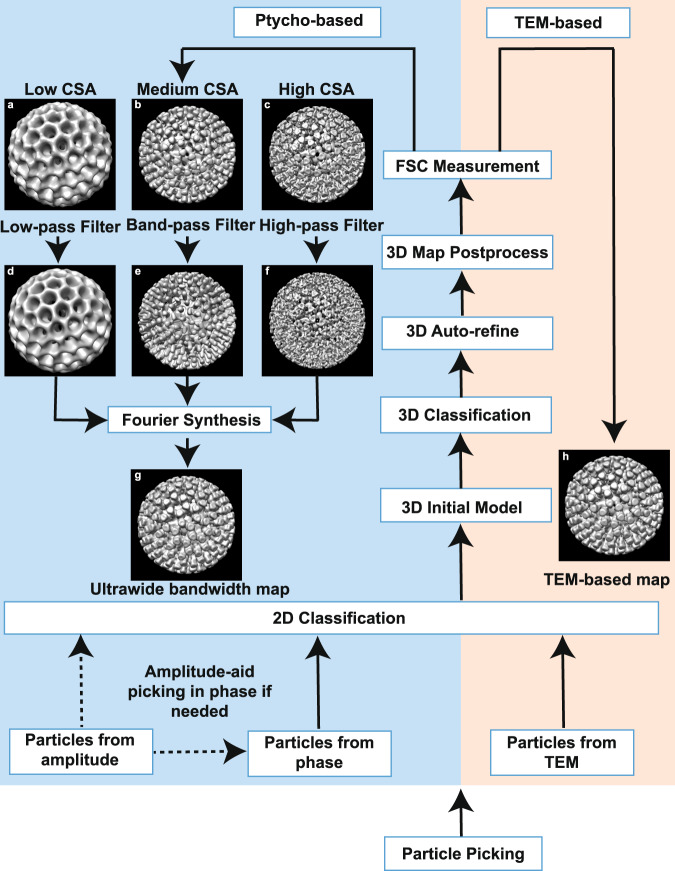
Comparison of workflows for Cryo EPty-SPA and TEM-SPA 3D reconstructions. **a**–**c** 3D electron density maps for low, medium, and high CSAs respectively. **d**–**f** Corresponding bandpass filtered maps of **a**–**c**, using the selected bandpass ranges where the information transfer is strongest. **g** Ultrawide bandwidth map obtained by Multi-band Fourier Synthesis (**h**) Band-limit map reconstructed by conventional TEM-SPA.

3D SPA reconstruction: An overall workflow as shown in Fig. 2, similar to that used in SPA can be applied to either the reconstructed phase or amplitude stacks using standard software packages such as Relion^7^ or EMAN^8^. It is important to note that contrast flipping to correct for the contrast transfer function (CTF) is not required as the ptychographic transfer function has no reversals^17^.

Multi-band Fourier synthesis: The bandwidth of information transfer for ptychography can be varied in the Fourier domain by changing the CSA^17^. Hence, multiple SPA 3D maps of the same sample with different spatial frequency bandwidths (low, medium and high frequencies as shown in Fig. 2a–c) can be obtained for different values of α. Furthermore, by combining the spatial frequencies most strongly transferred (Fig. 2d–f) in each SPA 3D map in the Fourier domain it is possible to generate a 3D map that contains a wider bandwidth of information transfer than that of any individual map as shown in Fig. 2g. We term this procedure Multi-band Fourier Synthesis (details are given in Methods).

### Cryo-ptychography with varying CSAs

**Fig. 3:**
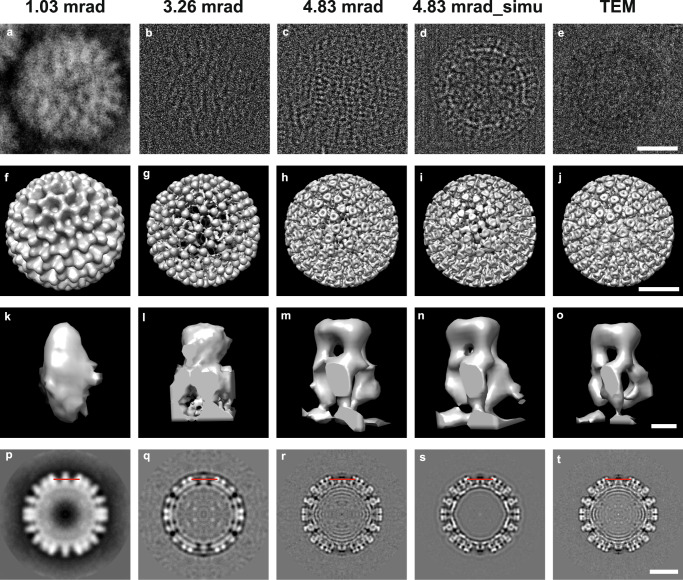
3D Rotavirus DLPs reconstructions using Cryo EPty-SPA for various CSAs and conventional TEM SPA. **a**–**c** Representative experimental ptychographic phase of a virus particle with CSAs, *α* = 1.03, 3.26 and 4.83 mrad from sets of typically 257, 443 and 498 reconstructed particles, respectively. **d** Representative simulated ptychographic phase of a particle for *α* = 4.83 mrad from a set of typically 305 simulated particles. **e** Representative TEM particle image from a set of typically 378 selected particles in ref. ^47^. **f**–**j** 3D maps refined with 232, 318, 241, 292 and 269 particles, respectively, corresponding to the particle instances (**a**–**e**). **k**–**o** Representative VP6 trimers selected from a total of 260 VP6 trimes in the outer shell of a rotavirus DLP and **p**–**t** central slices (the 125th slice from 248 slices in *z*-direction) extracted from the 3D maps in (**f**–**j**), respectively. Scale bars: 25 nm (**e**, **j**, **t**); 2.5 nm (**o**).

Following the protocol illustrated in Fig. 1, experimental cryo-EPty SPA datasets were recorded in different areas for vitrified samples of rotavirus double-layered viral particles (DLPs) using different values of the CSA, α. Single particle reconstructed phases for α = 1.03 mrad, 3.26 mrad, and 4.83 mrad (Fig. 3a–c) (corresponding amplitude data shown in Supplementary Fig. 2) were extracted from the ptychographic reconstructions using the ePIE^20^ algorithm (Supplementary Fig. 1) at doses of 22.6, 24.1, 24.6 e/Å^2^, respectively. For comparison, a previously published cryo-EM image of rotavirus DLPs recorded at a similar dose of 15 ~ 20 e/Å^2^ ^47^ is shown in Fig. 3e. At α = 1.03 mrad, the phase (Fig. 3a) shows strong low frequency transfer in the range from 0.014 to 0.116 nm^−1^ (8.6–72 nm) which defines the overall shape of the viral capsid proteins, consistent with our previous work^17^. As the CSA increased to 4.83 mrad (Fig. 3c), higher resoluion features of the viral capsid proteins are resolved.

To understand how frequency transfer is affected by α, it is necessary to examine the information transfer as a function of spatial frequency for ptychography. This determines how strongly each spatial frequency in the object is transferred, and hence the strength of the corresponding component in the final reconstruction. The transfer function calculated in previous work^17,48^ shows that for a given value of α the transfer function behaves as a bandpass filter that transfers components in a specified band of frequencies strongly. By varying α, this bandpass filter can be tuned to select either high or low periodicity features with no contrast reversals. This allows the reconstruction of multiple 3D maps from SPA data recorded with different values of the CSA which give rise to signal transfer in different bandwidths. These can subsequently be combined in the Fourier domain into a 3D map with a broad band of information transfer (See Methods). Details of the experimental settings for ptychographic data acquisition are provided in Supplementary Table 1 and Supplementary Text 1. Ptychographic reconstruction details are provided in Supplementary Text 2.

### Multi-band SPA reconstructions

Using the SPA pipeline (Fig. 2), three 3D density maps of rotavirus DLPs (Fig. 3f–h) were reconstructed from ptychographic phase data for different values of the CSA (calculation details are given in Supplementary Table 2 and Text 3), using the Relion3.1^7^ software package. The number of viral particles used for each map varied between 232 to 318, significantly less than that used for many conventional SPA reconstructions, although we note that icosahedral symmetry was imposed on the data. The low-angle 3D maps reconstructed from data at α = 1.03 mrad (Fig. 3f) and 3.26 mrad (Fig. 3g) clearly show the overall features of the outer VP6 capsid layer including 260 VP6 trimers (a typical VP6 trimer at 1.03 mrad and 3.26 mrad is shown in Fig. 3k and Fig. 3l). These are arranged in a T = 13 icosahedral lattice^47^, with the channels surrounded by the trimers. For α = 4.83 mrad, higher spatial frequency information is transferred^17^ such that finer details of the outer VP6 layer are visible in the 3D map (Fig. 3h). The central channels and three individual monomers can be directly visualised in the 3D volume of a trimer (Fig. 3m) extracted from the 3D map (Fig. 3h). It is also evident that the VP6 trimers are broader at the base than at the tip, and that each VP6 monomer follows an approximately helical path from the base inside the DLP to the tip of the trimer (Fig. 3m). These results are consistent with those from the cryo-EM SPA 3D map (Fig. 3j, o) and is further confirmed by line-profiles (Supplementary Fig. 3) extracted across a single VP6 trimer in the central slices of the 3D maps (Fig. 3p–t). The sharp valley in the blue line (Supplementary Fig. 3) indicates that the resolution at α=4.83 mrad is sufficient to resolve the channel in the trimer.

**Fig. 4:**
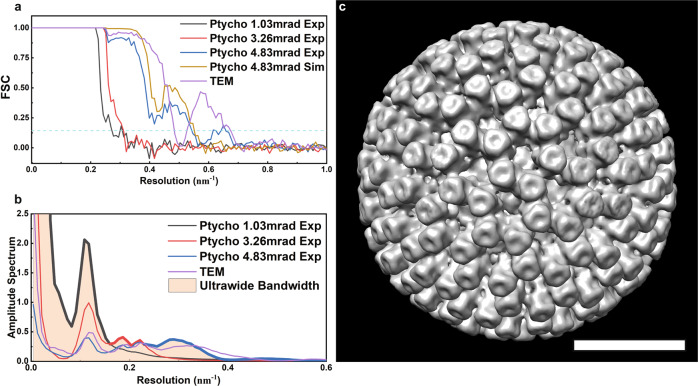
Fourier shell correlation, 3D amplitude spectrum and ultrawide bandwidth 3D map of Rotavirus DLPs. **a** Fourier shell correlation curves of the 3D maps in Fig. 3f–j. **b** Radially averaged amplitude spectra calculated from the 3D maps in Fig. 3f–h, j and Fig. 4(**c**). For each spectrum for a particular CSA value a bold line segment indicates the selected bandwidth within which the information transfer is strongest at that value of the CSA. For *α *= 1.03 mrad, the low-frequency bandwidth from 0~0.16 nm^−1^ (area 1 below the bold line segment in black) is selected. For *α* = 3.26 and 4.83 mrad, the selected bandwidths are at medium frequency from 0.17~0.23 nm^−1^ (area 2 below the bold segment in red) and high frequency from 0.23~0.76 nm^−1^ (area 3 below the bold segment in blue), respectively. **c** Ultrawide bandwidth 3D map obtained by multi-band Fourier synthesis from the 3D maps in Fig. 3f–h with *α* = 1.03 mrad (232 particles), 3.26 mrad (318 particles) and 4.83 mrad (241 particles), respectively. Scale bars: 25 nm (**c**).

The resolution of the 3D maps (Fig. 3f–h, j) are estimated as 3.72 nm, 3.29 nm, 1.86 nm, and 2.09 nm, respectively, using a Fourier Shell Correlation^49^ (FSC = 0.143, gold-standard). For simplicity, procedures^50^ to correct the effects of the CTF (for the TEM data) and MTF were not implemented in these reconstructions and we note that phase flipping is not required for the ptychographic phase. The 3D map calculated from the TEM data shows an oscillation in the FSC curve with a deep valley close to zero at 0.5 nm^−1^ (Fig. 4a), which is attributed to the zero-crossings and contrast reversal in the TEM CTF. These could however, be compensated in the 3D maps by implementation of CTF correction^11,51^, use of a wide range of defocus values^52^ and collection of data from a sufficiently large number of particles with varying defocus values. However, the FSC curve of the 3D maps using cryo-EPty SPA shows a gradual decrease with spatial frequency with no oscillations, due to the continuous positive nature of the ptychographic transfer function over its entire spatial frequency range^17^.

To validate this interpretation of our experimental results, cryo-EPty SPA simulations using the multislice method^53,54^ were carried out for a Rotavirus DLP model (3KZ4) using a software package described previously^55^ (for details of the calculations see Supplementary Table 3 and Text 4). When imaging frozen hydrated biological samples, a major resolution and contrast limiting factor is image blurring due to beam induced sample motion^56,57^, which in many instances cannot be prevented^58^. To model these effects, a range of empirical isotropic motion factors from 0-0.5 nm^57^ were included in the ptychographic simulations (see Supplementary Text 4). The simulated results (Supplementary Fig. 4) clearly show that the resolution (Supplementary Table 4) of the 3D maps for different CSAs were degraded as expected with increased motion factors.

For comparison with the experiment results (Fig. 3f–h), simulations (Supplementary Fig. 4) with various motion factors were calculated and the degree of fit between simulation and experiment was evaluated by normalized cross-correlation using the CHIMERA^59^ software (details are described in Supplementary Text 5). This fitting suggests that the motion factors present in the experimental data are in the range of 0.3–0.7 nm^57^ for the current experiments (Supplementary Fig. 5). Using α = 4.83 mrad as an example (Fig. 3i and Supplementary Fig. 6c), the VP6 trimers (Fig. 3n) extracted from the 3D map with an average motion factor of 0.5 ± 0.1 nm show similar features to those present in the experimental reconstruction (Fig. 3m). Line profiles (Supplementary Fig. 3) extracted from the central slices of the 3D map also show agreement between simulation and experiment. Furthermore, the FSC curves (Fig. 4a) indicate that the resolution of the simulated and experimental reconstructions are almost identical (1.83 nm vs 1.86 nm). Therefore, we conclude that the loss of resolution in the experiment with α = 4.83 mrad is equivalent to the effect of a 0.5 ± 0.1 nm beam-induced motion during acquisition as shown in Supplementary Table 4.

In conventional cryo-EM, the effects of beam-induced motion can be reduced or eliminated by optimizing the hole size of the perforated carbon films used for cryo-sample preparation^57^ or by using UltrAufoil grids^60^ or HexAu grids^61^ and recording movies of TEM images followed by drift correction to restore high-resolution information^62^ (as shown in Fig. 1i). Similarly, in ptychography, multi-frame diffraction patterns^32^ could be collected at each scanning position (Fig. 1c) followed by alignment of the patterns for motion correction. In future this approach will allow cryo-EPty SPA to achieve higher resolution.

To further explore the full potential of cryo-EPty SPA, a simulation with α = 15 mrad was calculated for a model of apoferritin (PDB-7A6A) without beam-induced motion. Supplementary Fig. 7a shows the resultant ptychographic phase of apoferritin and the corresponding 2D classifications, from which a 3D density map of apoferritin (Supplementary Fig. 7b) was reconstructed at a global resolution of 0.22 nm (Supplementary Fig. 7c) from 2826 particles at dose of 73.24 e/Å^2^, for a B-factor of −0.655556 nm^2^. An enlarged image (Supplementary Fig. 7d) extracted from the 3D map reveals backbone carbonyl groups, and distinct side-chain structural details including aromatic rings. This indicates that cryo-EPty SPA is capable of solving atomic structures of proteins and their ligand-bound complexes if larger values of α are used with sample motion correction and we note that the values of α required are easily obtained with C_s_ corrected optics.

### Wide bandwidth 3D map synthesis

Radial averages of 3D amplitude spectra were calculated (Fig. 4b) to directly compare the 3D information transfer as a function of spatial frequency for various values of the CSA. By tracing the intensity envelope of the amplitude spectra for the three values of the CSA used, the strongest information transfer bandwidth can be identified for each. For example, the 3D map for α = 1.03 mrad has strongest transfer in a low-frequency bandwidth (area 1 below the bold line segment in black as shown in Fig. 4b). The bandwidths with the strongest information transfer at different values of the CSA are identified in Fig. 4b and their ranges are given in Supplementary Table 5. Using this information, the 3D maps from datasets with different values of the CSA were then band-pass filtered using the corresponding selected bandwidths, giving band-pass filtered 3D maps (Supplementary Fig. 8), each of which, individually gives the best information transfer within a given spatial frequency bandwidth. In summary our approach is to choose, for any given spatial frequency, the data from the CSA that has the strongest signal for that frequency and to discard the weaker signals from other CSA data. Although this is not an optimally efficient reconstruction strategy since some data from any one experiment is discarded, for the case of SPA reconstruction adding additional data does not increase the dose of any one particle and each dataset is recorded from a different set of particles.

By combining these filtered 3D maps (Supplementary Fig. 8), a wide bandwidth 3D map (Fig. 4c) can be synthesised using the procedure illustrated in Fig. 2. Compared to the 3D maps using a dataset with a single value of α (Fig. 3f–h), the wide bandwidth 3D map (Fig. 4c) contains strong information transfer across a wider range of spatial frequencies (shaded beige in Fig. 4b) and hence provides improved transfer of both low and high frequency information (Supplementary Table 5). This conclusion is further supported by simulations (Supplementary Fig. 9).

## Discussion and conclusions

We have demonstrated cryo-EPty SPA for reconstruction of rotavirus particles both experimentally and with simulations using the protocol shown in Figs. 1 and 2. It is therefore useful to discuss the differences in SPA reconstructions using different input data, specifically comparing the use of defocused phase contrast TEM images and reconstructed ptychographic phases.

In terms of the respective optical configurations, the major difference is that TEM uses static plane wave illumination (Fig. 1h), whereas a convergent beam is scanned across the sample during ptychographic data acquisition (Fig. 1a). Moreover as ptychography records diffraction patterns, the objective lens aberrations are not important in determining the information transfer. Ultimately, for this reason, ptychography can theoretically reach a resolution set by the diffraction limit as has been demonstrated for radiation resistant samples^24,63^.

For TEM data (Fig. 1i), the FOV and the pixel sampling in an image must be balanced due to the finite pixel size and array size of imaging detectors (typically a maximum of ca. 4096 × 4096 pixels) and consequently there is a trade-off between resolution and FOV (e.g. a sampling of 0.1 nm per pixel leads to approximately a 400 nm FOV for a typical 4k × 4k detector). For a given detector size, finer sampling must be used, at higher magnifications, resulting in a reduction in the number of particles imaged in each frame for a smaller FOV^64^. In contrast, for ptychography the sampling of the image is limited by the highest angle collected by the detector. Therefore, even when using smaller detectors (256 × 256 Merlin Medipix3^34^ and 128 × 128 EMPAD^35^) ptychography can still obtain an unlimited FOV at high resolution if sufficient probe positions are recorded.

Cryo-EM SPA can be used to fine tune the low order aberrations before image acquisition (Fig. 1h), usually in an adjacent area to the target area. In contrast ptychographic reconstruction algorithms can reconstruct both probe and object functions^20^ and compensate for residual low order aberrations after data acquisition^28,29^. This is particularly important at low dose where accurate determnination of the aberrations during acquisition is challenging due to a low SNR.

The ptychographic transfer function leads to reconstructions that are free of contrast reversals over the entire spatial frequency range recovered. As a result, there is no need for phase reversal correction during SPA reconstruction as is routinely used in cryo-EM SPA (Fig. 1j–k). Furthermore, as we have shown, the ptychographic transfer function can also be adjusted by changing the CSA of the probe^17^, giving multiple reconstructions with different optimal contrast transfer bandwidths. Subsequently, using a multi-band Fourier synthesis as described here, a 3D map with high signal transfer is obtained over a wide spatial frequency range. Similarly, TEM SPA can also combine multiple defocus values to provide sufficient contrast for selection (high defocus) and sufficient resolution (low defocus). Although, as already described some data is discarded in the process described in the case of SPA reconstructions this is acceptable as more images can be added without increasing the dose of any one particle.

Furthermore, we emphasise that the complex wavefunction (both phase and amplitude) of an object is recovered using ptychography, which provides additional information for particle picking or model initialization in SPA. Finally, ptychography using an inverse multi-slice method^29^ can computationally section z-slices of interest from thicker biological samples, where the projection approximation breaks down as a consequence of significant multiple scattering within the sample.

However, there are still experimental challenges, mainly related to beam-induced motion which limits the achievable resolution for ptychographic SPA reconstruction of biological samples embedded in vitrified ice. The first is to eliminate or reduce the sample drift. Considering the various possible mechanisms of beam-induced motion, such as a build-up of charge^65^, radiolysis of the specimen and mechanical stress in the ice layer and carbon support^66,67^, methods have been proposed to overcome these and to mitigate the effects of motion^57^. An alternative is to implement drift correction in the ptychographic data acquisition. For this, a movie of diffraction patterns needs to be recorded at each position and a drift-correction algorithm, adapted to ptychographic iterative reconstruction will be required to reduce the effect of the motion blur. The third possibility is to outrun the sample motion by recording data at high spatio-temporal resolution. For example, an ultra-fast detector with a much high frame rate, such as 10,000 fps^68^ (1000 fps used in our work) has been developed. This together with a pulsed electron source, potentially allows the data acquisition to outrun the sample motion and avoiding blurring effects^69^.

In summary, we have described a workflow for high contrast, wide spatial frequency-band SPA reconstructions which could be extended to higher resolution. With improved microscope stability, improved reconstruction algorithms (machine learning) specifically optimized for low signal data^70^, ultrafast detectors, and pulsed electron sources, ptychography has potential application for structure determination with some advantages over the use of conventional phase contrast images.

## Methods

### Samples

A suspension of Rotavirus DLPs (strain SA11) was prepared using a previously described method^17^. The DLPs/AuNPs suspension (4 μl) was placed onto holey carbon EM grids (Quantifoil). Each grid was blotted for 5 s and subsequently plunged into a liquid ethane/propane mixture cooled by liquid nitrogen at 80% humidity. The grids were then transferred and stored under liquid nitrogen. Further details of the sample preparation are provided in Supplementary Text 1.

### Cryo-ptychography experiment setup

The cryo-EPty experiments were performed in a scanning diffraction mode on a JEOL ARM 300CF operated at 300 kV with data recorded on a pixelated Merlin Medipix3 detector^34^. The vitrified EM samples were transferred into a Gatan 698 Elsa cryo-transfer holder to ensure that the specimen temperature was maintained at −176 ± 2 °C. Three probe CSAs of 1.03 mrad, 3.26 mrad, and 4.83 mrad were used to record ptychographic datasets. Detailed additional experimental parameters for these values of the CSA are given in Supplementary Table 1 and Supplementary Text 1.

### ePIE reconstructions

Ptychographic reconstructions were carried out using the ePIE^20^ algorithm run for 100 iterations. Detailed reconstruction parameters for each value of α are given in Supplementary Table 1 and Supplementary Text 2. The degree of redundancy was estimated as 217, 336, and 155 for the datasets at α values of 1.03, 3.26 and 4.83 mrad, respectively. Details of the redundancy calculation used are provided in Supplementary Text 6. The computational time cost for ptychographic data collection and processing is described in Supplementary Text 7.

### Single particle reconstruction

Using the e2box.py program in EMAN2.2^8^, 257, 443, 498, and 378 particles were picked for α = 1.03 mrad, 3.26 mrad, and 4.83 mrad and TEM, respectively. Subsequently, the 3D reconstruction workflow as described was implemented using the software package Relion 3.1^7^. Further details are provided in Supplementary Text 3.

### Ptychographic simulations

Multislice simulations of cryo-EPty SPA reconstructions using models of rotavirus DLPs and apoferritin used were carried out using the multislice algorithm^53,54^ implemented in the Matlab code InSilicoTEM^55^. The atomic potential maps of rotavirus DLPs and apoferritin were built from the 3KZ4 and 7A6A models in the protein data bank. Detailed simulation parameters and related information for various CSA values are given in Supplementary Table 3 and Supplementary Text 4.

### Multi-band SPA reconstructions

We define ${\{{\alpha }_{i}\}}_{i=1}^{n}=\{{\alpha }_{1},{\alpha }_{2},{\alpha }_{3},\ldots,{\alpha }_{n}\}$ as a set of *n* different values of α for which a corresponding set of 3D electron density maps $\{{V}_{{\alpha }_{i}}(\mathop{{{{\bf{r}}}}}\limits^{{\rightharpoonup }})\}_{i=1}^{n}=\{{V}_{{\alpha }_{1}}(\mathop{{{{\bf{r}}}}}\limits^{{\rightharpoonup }}),\,{V}_{{\alpha }_{2}}(\mathop{{{{\bf{r}}}}}\limits^{{\rightharpoonup }}),{V}_{{\alpha }_{3}}(\mathop{{{{\bf{r}}}}}\limits^{{\rightharpoonup }}),\ldots,{V}_{{\alpha }_{n}}(\mathop{{{{\bf{r}}}}}\limits^{{\rightharpoonup }})\}$ were reconstructed, with $\mathop{{{{\bf{r}}}}}\limits^{{\rightharpoonup }}$ a 3D vector in real space. The amplitude spectra in 3D are given as the modulus of the Fourier transforms of the 3D electron density maps ${W}_{{\alpha }_{i}}(\mathop{{{{\bf{q}}}}}\limits^{{\rightharpoonup }})=ft({V}_{{\alpha }_{i}}(\mathop{{{{\bf{r}}}}}\limits^{{\rightharpoonup }}))$ forming a set of $\{{W}_{{\alpha }_{i}}(\mathop{{{{\bf{q}}}}}\limits^{{\rightharpoonup }})\}_{i=1}^{n}=\{{W}_{{\alpha }_{1}}(\mathop{{{{\bf{q}}}}}\limits^{{\rightharpoonup }}),\,{W}_{{\alpha }_{2}}(\mathop{{{{\bf{q}}}}}\limits^{{\rightharpoonup }}),{W}_{{\alpha }_{3}}(\mathop{{{{\bf{q}}}}}\limits^{{\rightharpoonup }}),\ldots,\,{W}_{{\alpha }_{n}}(\mathop{{{{\bf{q}}}}}\limits^{{\rightharpoonup }})\}$, with $\mathop{{{{\bf{q}}}}}\limits^{{\rightharpoonup }}$ a 3D vector in Fourier space.

To evaluate the information transfer of ${W}_{{\alpha }_{i}}(\mathop{{{{\bf{q}}}}}\limits^{{\rightharpoonup }})$, we calculate one-dimensional radially averaged amplitude spectra ${l}_{{\alpha }_{i}}(q)$ for each *α*_*i*_ as a function of a radius, *q* in 3D Fourier space as shown in Fig. 4b. The amplitude spectra are expressed as:

1 $${l}_{{\alpha }_{i}}(q)=\frac{{\sum }_{q-\frac{b}{2} < |\mathop{{{{\bf{q}}}}}\limits^{{\rightharpoonup }}|\le q+\frac{b}{2}}|{W}_{{\alpha }_{i}}(\mathop{{{{\bf{q}}}}}\limits^{{\rightharpoonup }})|}{{\sum }_{q-\frac{b}{2} < |\mathop{{{{\bf{q}}}}}\limits^{{\rightharpoonup }}|\le q+\frac{b}{2}}1}$$

where ${\sum }_{q-\frac{b}{2} < |\mathop{{{{\bf{q}}}}}\limits^{{\rightharpoonup }}|\le q+\frac{b}{2}}|{W}_{{\alpha }_{i}}(\mathop{{{{\bf{q}}}}}\limits^{{\rightharpoonup }})|$ is the sum of the modulus of ${W}_{{\alpha }_{i}}(\mathop{{{{\bf{q}}}}}\limits^{{\rightharpoonup }})$ with a bin width, *b* at a radius, *q* and ${\sum }_{q-\frac{b}{2} < |\mathop{{{{\bf{q}}}}}\limits^{{\rightharpoonup }}|\le q+\frac{b}{2}}1$ is the total number of voxels within this bin width. From this, a set of radially averaged amplitude spectra $\{{l}_{{\alpha }_{i}}(q)\}_{i=1}^{n}=\{{l}_{{\alpha }_{1}}\left(q\right),{l}_{{\alpha }_{2}}\left(q\right),{l}_{{\alpha }_{3}}\left(q\right),\ldots,{l}_{{\alpha }_{n}}\left(q\right)\}$ can be obtained.

Each spectrum corresponding to a particular value, *α*_*i*_ has a bandwidth ${B}_{{\alpha }_{i}}$ (a segment in *q* space as shown in Fig. 4b), within which the strength of the information transfer ${l}_{{\alpha }_{i}}(q)$ is strongest. The bandwidth for *α*_*i*_ is given as:

2 $${B}_{{\alpha }_{i}}=\{q|{l}_{{\alpha }_{i}}(q)\ge {l}_{{\alpha }_{j}}(q),\;\; for \quad \forall j \, \ne \, i\}$$

which forms a set of bandwidths ${\{{B}_{{\alpha }_{i}}\}}_{i=1}^{n}=\{{B}_{{\alpha }_{1}},\,{B}_{{\alpha }_{2}},\,{B}_{{\alpha }_{3}},\ldots,\,{B}_{{\alpha }_{n}}\}$.

To carry out the multi-band Fourier synthesis, the signals most strongly transferred in each element of the Fourier amplitude spectrum set $\{{W}_{{\alpha }_{i}}(\mathop{{{{\bf{q}}}}}\limits^{{\rightharpoonup }})\}_{i=1}^{n}$ are assigned to ${W}_{\max }(\mathop{{{{\bf{q}}}}}\limits^{{\rightharpoonup }})$ subject to the condition that $|\mathop{{{{\bf{q}}}}}\limits^{{\rightharpoonup }}|$ is within the corresponding element of the bandwidth set ${\{{B}_{{\alpha }_{i}}\}}_{i=1}^{n}$ given by:

3 $${W}_{\max }(\mathop{{{\bf{q}}}}\limits^{{\rightharpoonup }})	=\left\{\begin{array}{ccc}{W}_{{\alpha }_{1}}(\mathop{{{\bf{q}}}}\limits^{{{\rightharpoonup }}})& {\,}&|\mathop{{{{\bf{q}}}}}\limits^{{{\rightharpoonup }}}|\in {B}_{{\alpha }_{1}}\\ {W}_{{\alpha }_{2}}(\mathop{{{{\bf{q}}}}}\limits^{{{\rightharpoonup }}})&{\,} &|\mathop{{{{\bf{q}}}}}\limits^{{{\rightharpoonup }}}|\in {B}_{{\alpha }_{2}}\\ & \vdots & \\ {W}_{{\alpha }_{n}}(\mathop{{{{\bf{q}}}}}\limits^{{{\rightharpoonup }}}){\,} & {\,} &|\mathop{{{{\bf{q}}}}}\limits^{{{\rightharpoonup }}}|\in {B}_{{\alpha }_{n}}\end{array} \right.\\ 	=\,\max (\{{W}_{{\alpha }_{i}}(\mathop{{{{\bf{q}}}}}\limits^{{\rightharpoonup }})\}_{i=1}^{n})$$

The multi-band Fourier synthesis 3D map ${V}_{\max }(\mathop{{{{\bf{r}}}}}\limits^{{\rightharpoonup }})$ can then be generated by 3D inverse Fourier transforming ${W}_{\max }(\mathop{{{{\bf{q}}}}}\limits^{{\rightharpoonup }})$ to give:

4 $${V}_{\max }(\mathop{{{{\bf{r}}}}}\limits^{{\rightharpoonup }})=f{t}^{-1}({W}_{\max }(\mathop{{{{\bf{q}}}}}\limits^{{\rightharpoonup }}))$$

which contains a much wider bandwidth and stronger information transfer than those of any individual element in the 3D map set $\{{V}_{{\alpha }_{i}}(\mathop{{{{\bf{r}}}}}\limits^{{\rightharpoonup }})\}_{i=1}^{n}$.

### Reporting summary

Further information on research design is available in the Nature Portfolio Reporting Summary linked to this article.

## Supplementary information

Supplementary Information

Reporting Summary

## Data Availability

The data that support this study are available from the corresponding authors upon request. The raw data (diffraction datasets) in this study have been deposited in the Zenodo database [10.5281/zenodo.7701710]. The cryo-EPty maps have been deposited in the Electron Microscopy Data Base (EMDB) under accession codes EMD-35828 (CSA 1.03 mrad), EMD-35916 (CSA 3.26 mrad) and EMD-35917 (CSA 4.83 mrad). The reconstructed phase image data are available as EMPIAR datasets under accession codes EMPIAR-11500 (CSA 1.03 mrad), EMPIAR-11503 (CSA 3.26 mrad), and EMPIAR-11504 (CSA 4.83 mrad). Previously published structural models can be accessed using PDB accession codes 3KZ4 and 7A6A.

## Code availability

Distributions of ePIE code are subject to an agreed licence agreement with Phase Focus Ltd. and the authors of this paper are not permitted to distribute the ePIE code used under the terms of that agreement. The EMAN2 code is available as open source under a General Public License (GPL) version v2.0 from the authors [https://github.com/cryoem/eman2]. The Relion3 code is available as open source under a GNU General Public licence v2.0 [https://github.com/3dem/relion].
