# Supplementary material for: Cryogenic electron ptychographic single particle analysis with wide bandwidth information transfer
Source: Nat Commun. 2023 May 25;14:3027. doi: 10.1038/s41467-023-38268-0 (PMC10212999; doi:10.1038/s41467-023-38268-0)
Supplement: Supplementary file 1 — Supplementary Information [file 41467_2023_38268_MOESM1_ESM.pdf]

Supplementary Information

Cryogenic Electron Ptychographic Single Particle Analysis with Wide Bandwidth Information Transfer

Xudong Pei, Liqi Zhou, Chen Huang et al.

Supplementary Figures

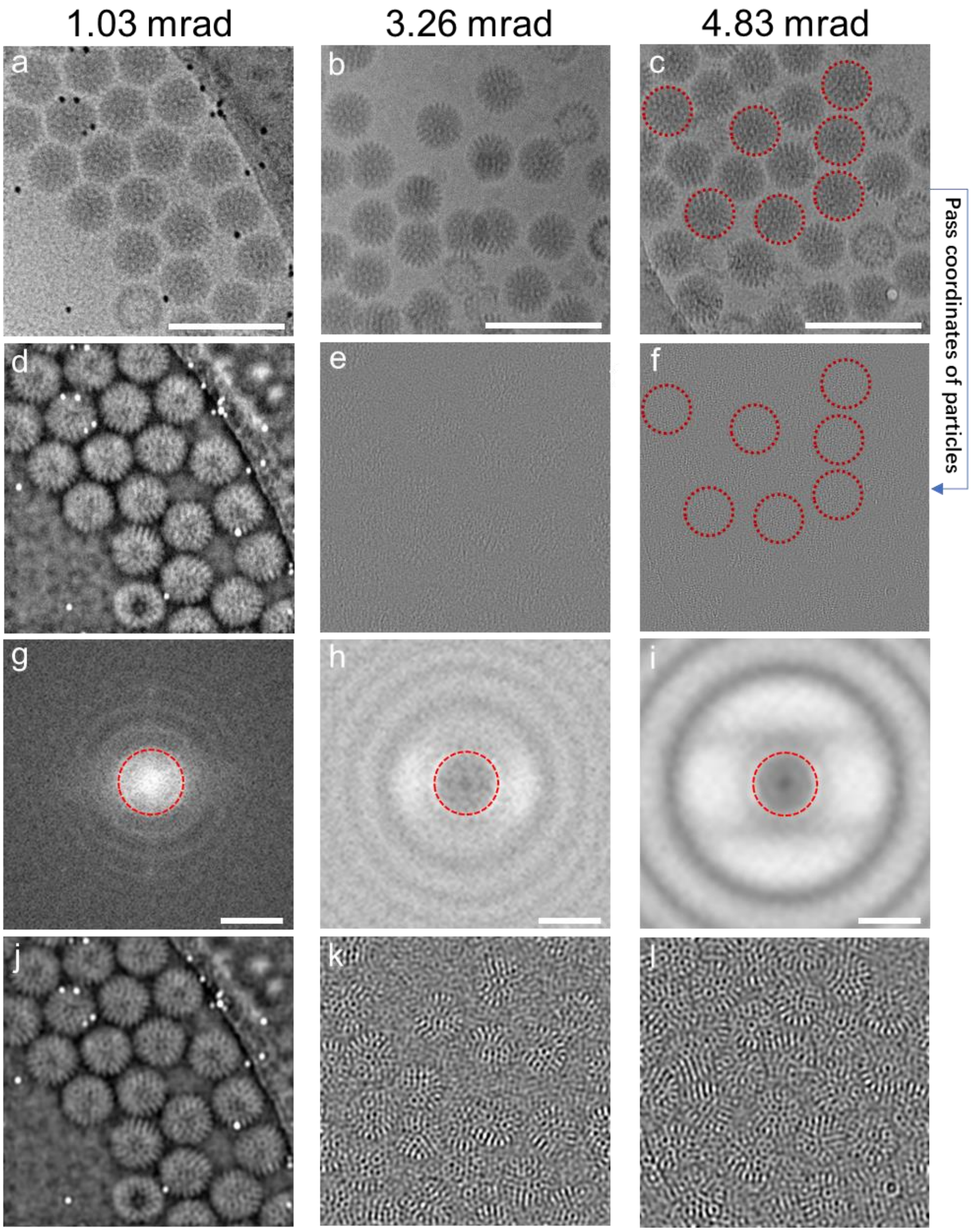

Supplementary Figure 1

Representative ptychographic reconstructions of rotavirus DLPs in vitrified ice at a similar dose of ~ 23 e/Å^2 at various convergence semi angles(CSAs) (Left column: 1.03 mrad; Middle column: 3.26 mrad; Right column: 4.83 mrad) selected from the 29, 22 and 22 reconstructed datasets, respectively. (**a** to **c**) Amplitude, (**d** to **f**) phase, (**g** to **i**) corresponding amplitude spectra of the phases calculated from (**d** to **f**), (**j** to **l**) filtered phases with a low-pass filter from 0 to 0.116 nm^{-1} indicated with a red circle in (**g** to **i**). Scale bars in (**a** to **c**): 160 nm. Scale bars in (**g** to **i**): 0.232 nm^{-1}.

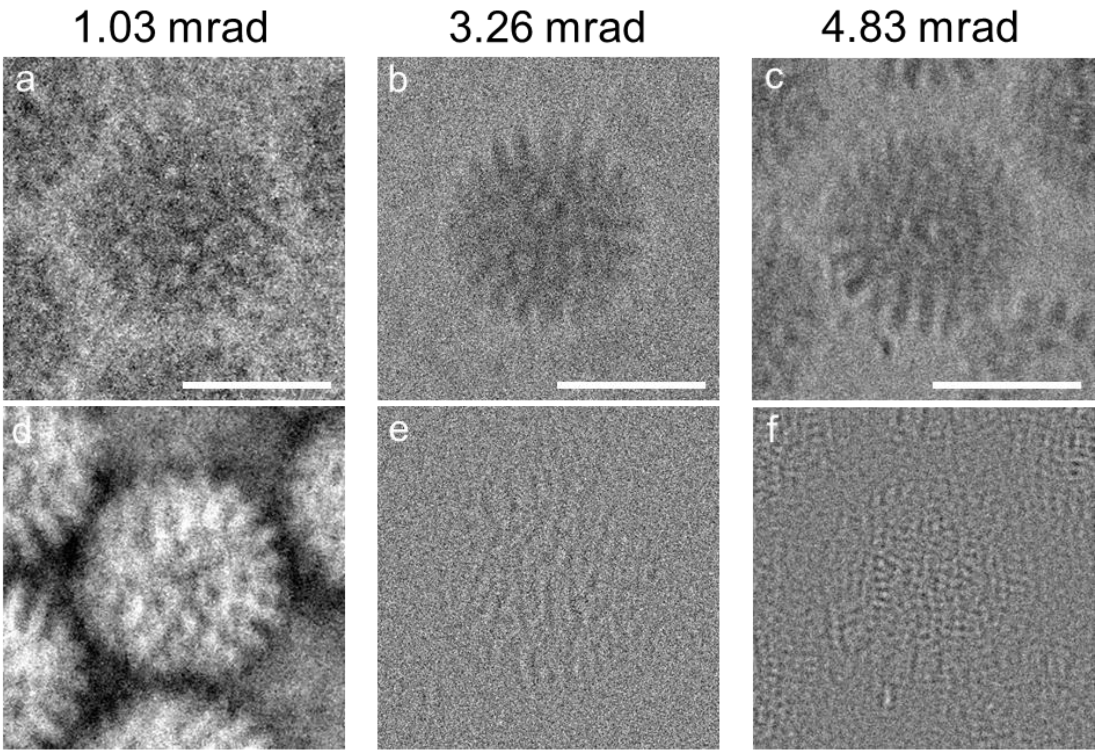

Supplementary Figure 2

Representative ptychographic reconstructions of rotavirus DLPs in vitrified ice at a similar dose of ~ 23 e/Å^2 at various CSAs (Left column: 1.03 mrad; Middle column: 3.26 mrad; Right column: 4.83 mrad) extracted from the sets of typically 257, 443 and 498 reconstructed particles, respectively. (**a** to **c**) Amplitude, (**d** to **f**) phase of the reconstructions. Scale bars: 50 nm.

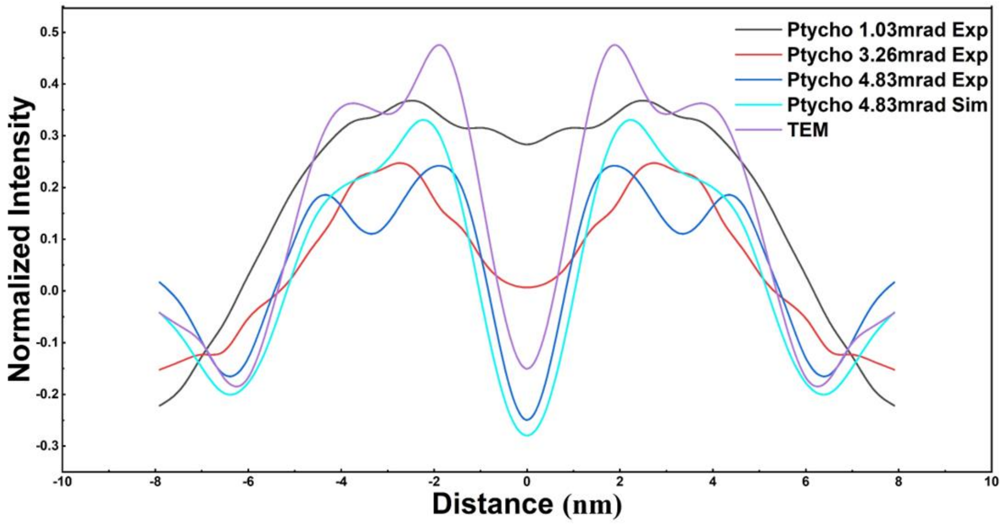

Supplementary Figure 3

Line profiles extracted from Fig. 3 (**p** to **t**) (main Text) at the position indicated with a red line.

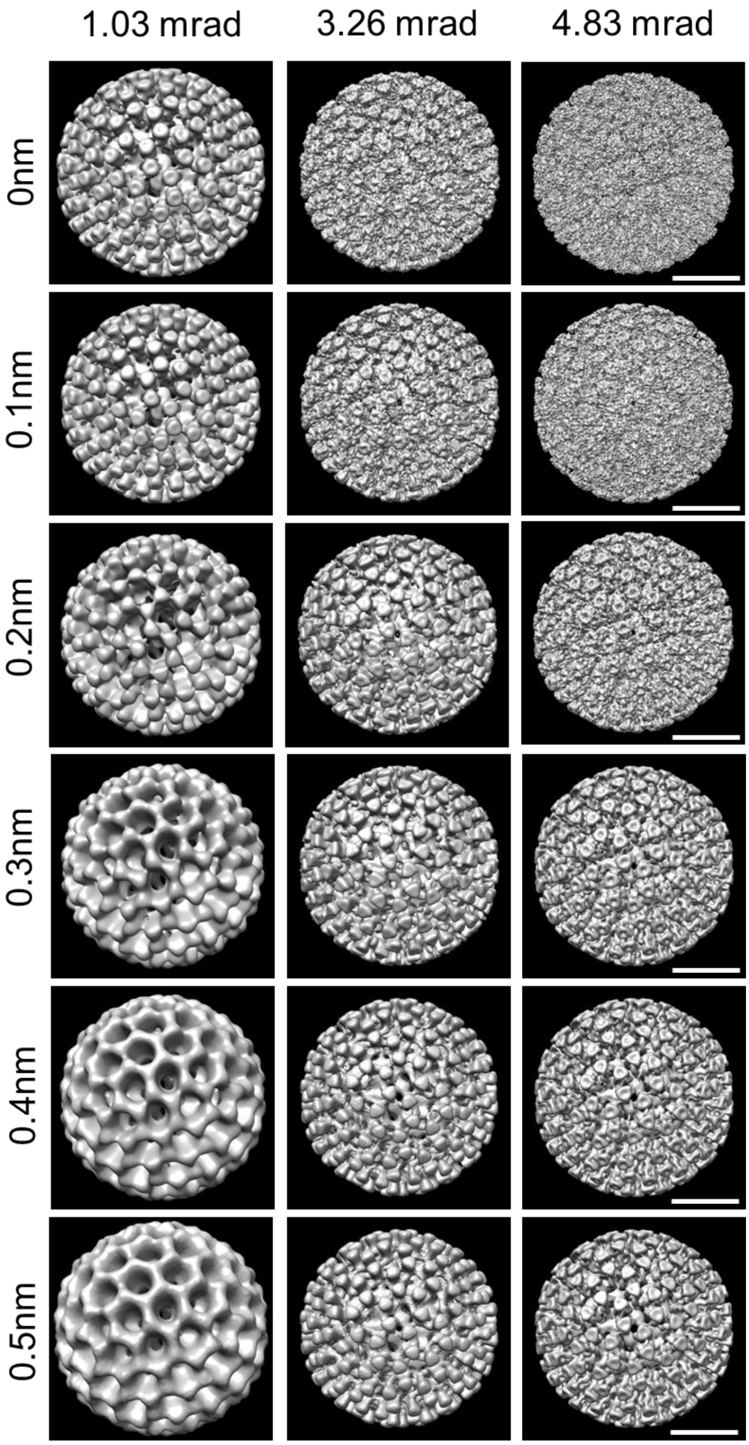

Supplementary Figure 4

Simulated ptychographic SPA 3D reconstructions; Left column: 1.03 mrad (refined with 292, 283, 351, 352, 319 and 347 particles for different motion blur factors from 0 to 0.5nm, respectively); Middle column: 3.26 mrad (refined with 289, 289, 289, 289, 287 and 289 particles for motion blur factors from 0 to 0.5nm, respectively); Right column: 4.83 mrad (refined with 305, 305, 305, 304, 301 and 292 particles for motion blur factors from 0 to 0.5nm, respectively). Scale bars: 25 nm.

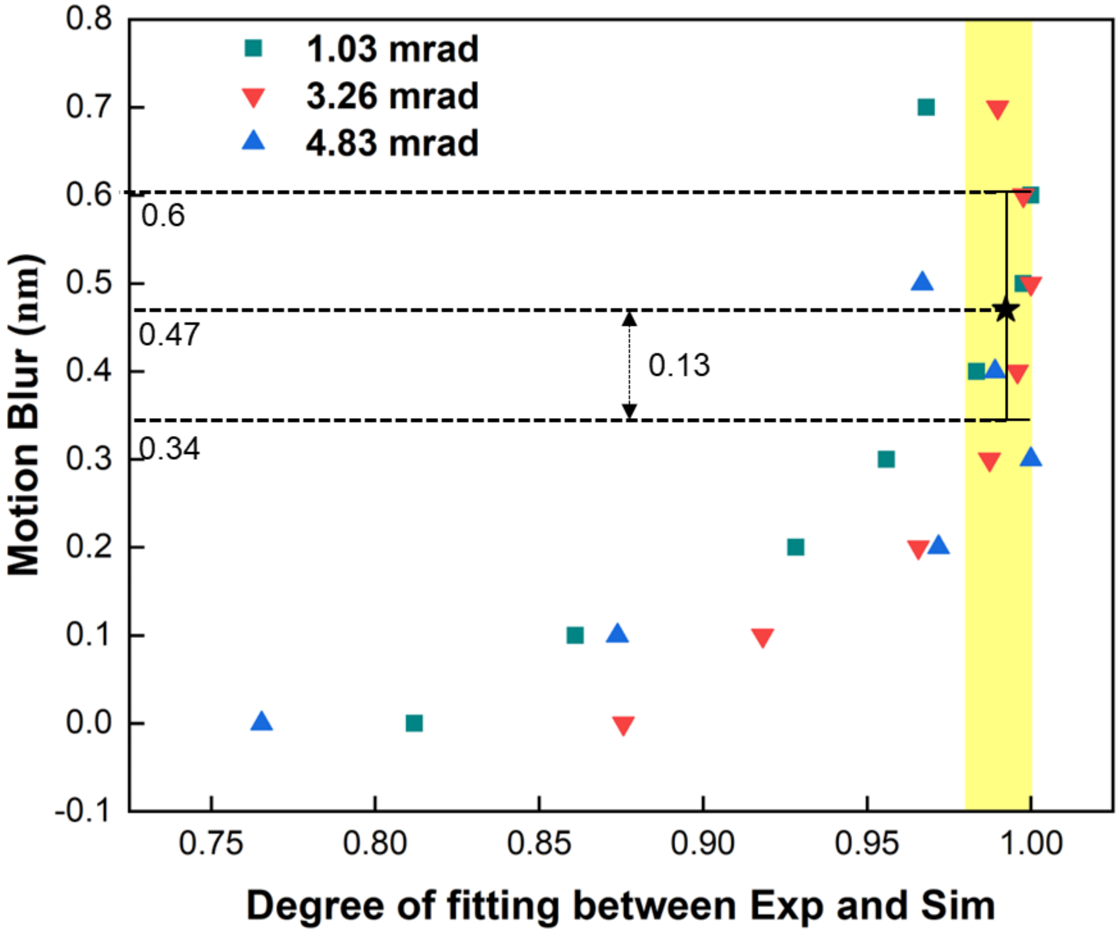

Supplementary Figure 5

Normalised degree of fitting between experimental and simulated maps for α=1.03 mrad, 3.26 mrad and 4.83 mrad as a function of motion factors ranging from 0-0.7 nm. In total, 22 independent evaluations were performed for all three angles and various motion factors. Among these evaluations, 10 data points of the fitting values were found to be greater than 98% within the yellow region. Based on these data points, an average motion factor of 0.47 nm and a standard deviation of 0.13 nm were calculated for the current experimental system. The degree of fitting was evaluated by normalised cross-correlation using the CHIMERA[1] software. (Further details are described in Supplementary Note 5).

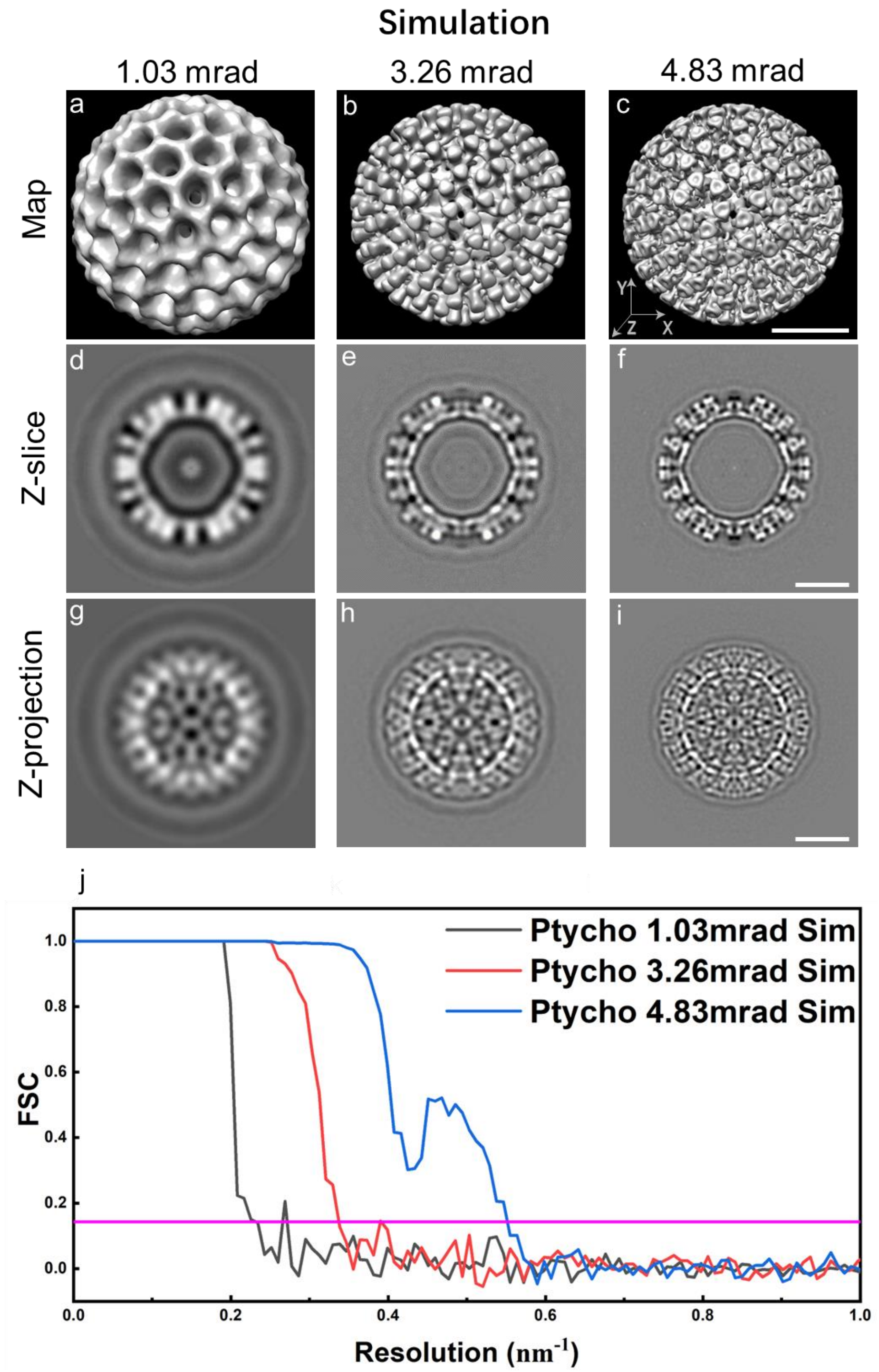

Supplementary Figure 6

Simulated ptychographic SPA reconstructions with the same CSA values and similar dose to those used in the experimental data in Fig. 3 (**f** to **h**) (main Text). (**a** to **c**) 3D electron density maps at CSAs of 1.03 mrad refined with 347 particles (Left column), 3.26 mrad refined with 289 particles (Middle column) and 4.83 mrad refined with 292 particles (Right column) with their corresponding central slices (the 125th slice from total 248 slices in z-direction) (**d** to **f**) and z-direction projections (**g** to **i**). (**j**) FSC curves calculated from (**a** to **c**). Scale bars: 25 nm.

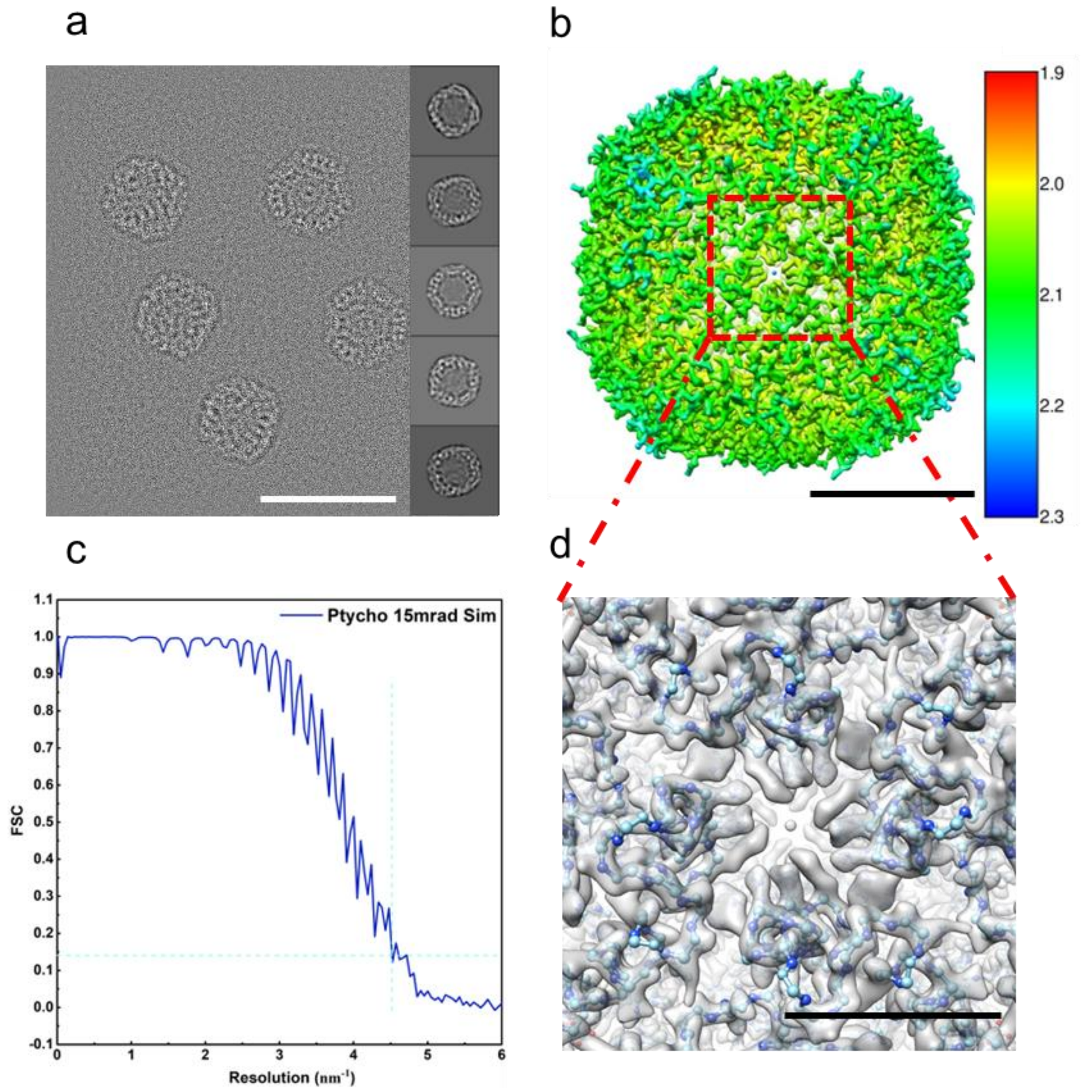

Supplementary Figure 7

Simulated Cryo EPty-SPA reconstruction of apoferritin at atomic resolution. (**a**) Representative simulation of the ptychographic phase selected from a set of typically 542 simulated datasets and 2D classification. (**b**) 3D map refined with 2826 particles. (**c**) Fourier shell correlation curve of the 3D map in (**b**). (**d**) 3D enlarged map fitted with the model of apoferritin of the area indicated with a red dashed square in (**b**). Scale bars: 20 nm (**a**); 5 nm (**b**); 2 nm (**d**).

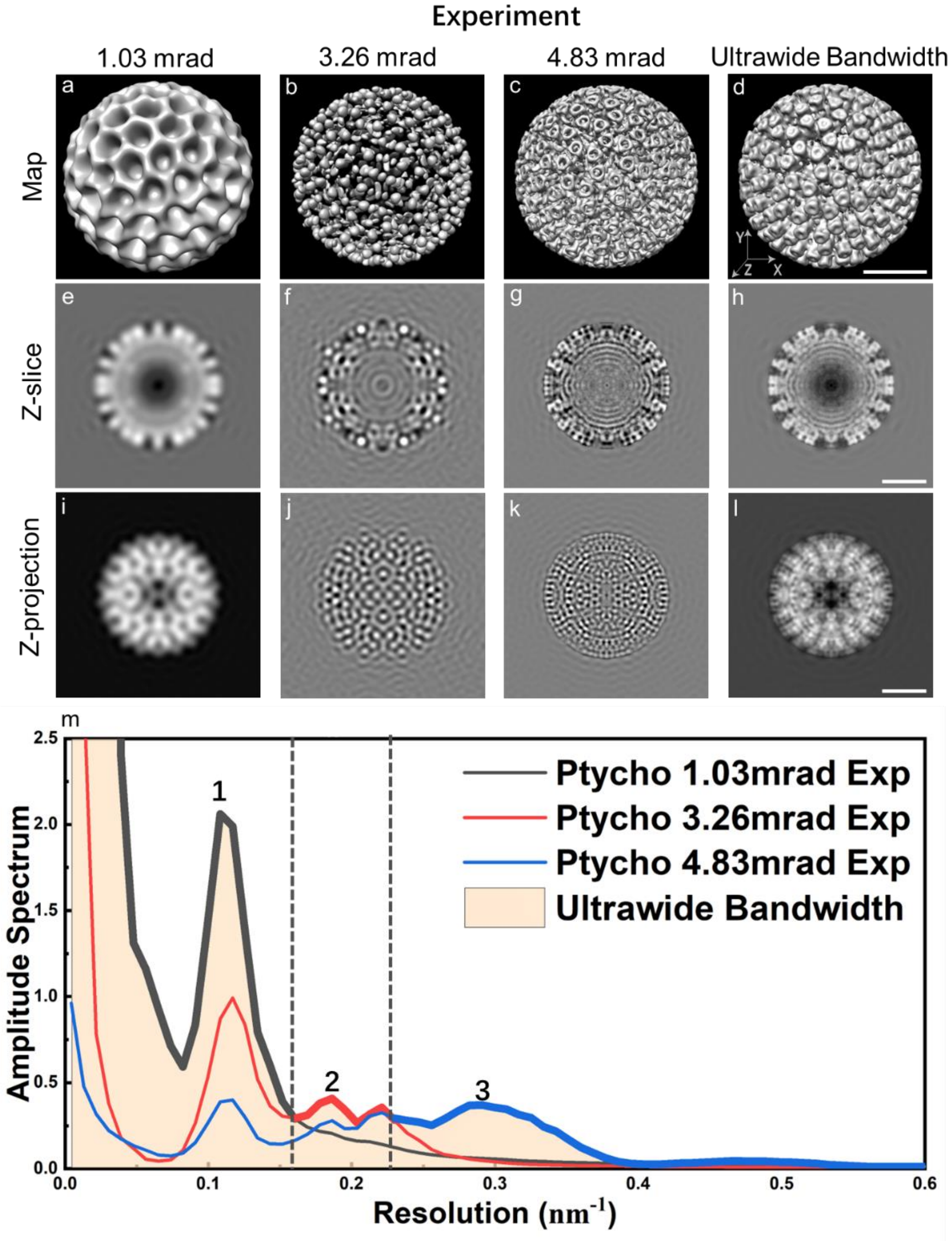

Supplementary Figure 8

Multi-band Fourier synthesis of experimental ptychographic SPA 3D maps**.** Dominant band-pass filtered 3D maps from 0 nm^{-1} to 0.1691 nm^{-1} for α=1.03 mrad (**a**), from 0.1691 nm^{-1} to 0.2298 nm^{-1} for α=3.26 mrad (**b**), and from 0.2298 nm^{-1} to 0.76 nm^{-1} for α=4.83 mrad (**c**). Synthesised 3D map (**d**) with wide bandwidth information transfer obtained by combining the information transferred in the Fourier domain of (**a** to **c**). Corresponding central slices (the 125th slice from total 248 slices in z-direction) (**e** to **h**) and z-direction projections (**i** to **l**) for (**a** to **d**). (**m**) Radially averaged amplitude spectra calculated from (**a** to **d**). For each spectrum, a bold line segment indicates the dominant bandwidth, within which information transfer is strongest. Scale bars: 25 nm.

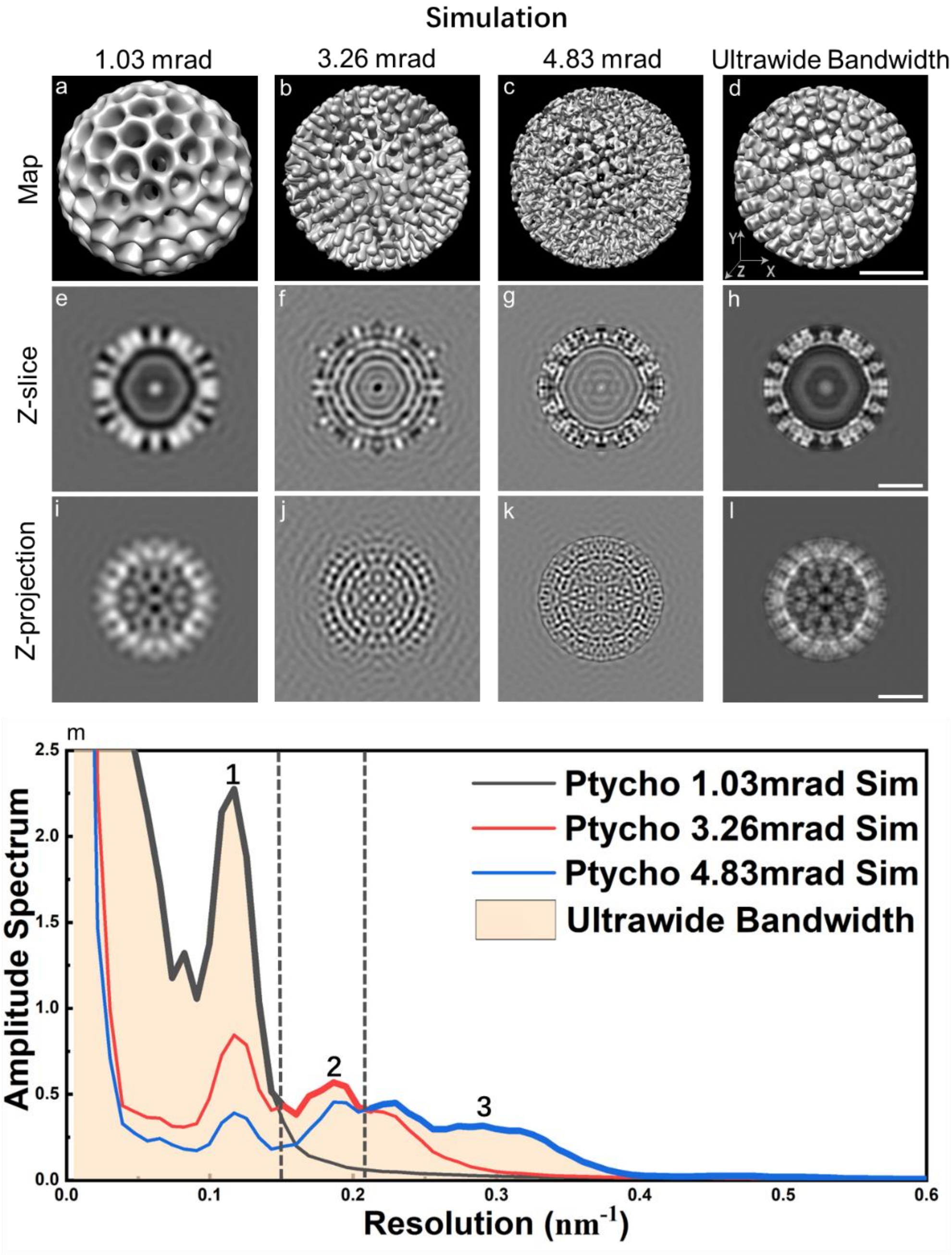

Supplementary Figure 9

Multi-band Fourier Synthesis of simulated ptychographic SPA 3D maps**.** Dominant band-pass filtered 3D maps from 0 nm^{-1} to 0.1518 nm^{-1} for α=1.03 mrad (**a**), from 0.1518 nm^{-1}~0.2129 nm^{-1} for α=3.26 mrad (**b**), and from 0.2129 $nm^{-1}$to 0.76 nm^{-1} for α=4.827 mrad (**c**). Synthesised 3D map (**d**) with a wide bandwidth information transfer obtained by combining the information transferred in the Fourier domain of (**a** to **c**). Corresponding central slices (the 125th slice from total 248 slices in z-direction) (**e** to **h**) and z-direction projections (**i** to **l**) for (**a** to **d**). (**m**) Radially averaged amplitude spectra calculated from (**a** to **d**). For each spectrum a bold line segment indicates the dominant bandwidth, within which the information transfer is strongest. Scale bars: 25 nm.

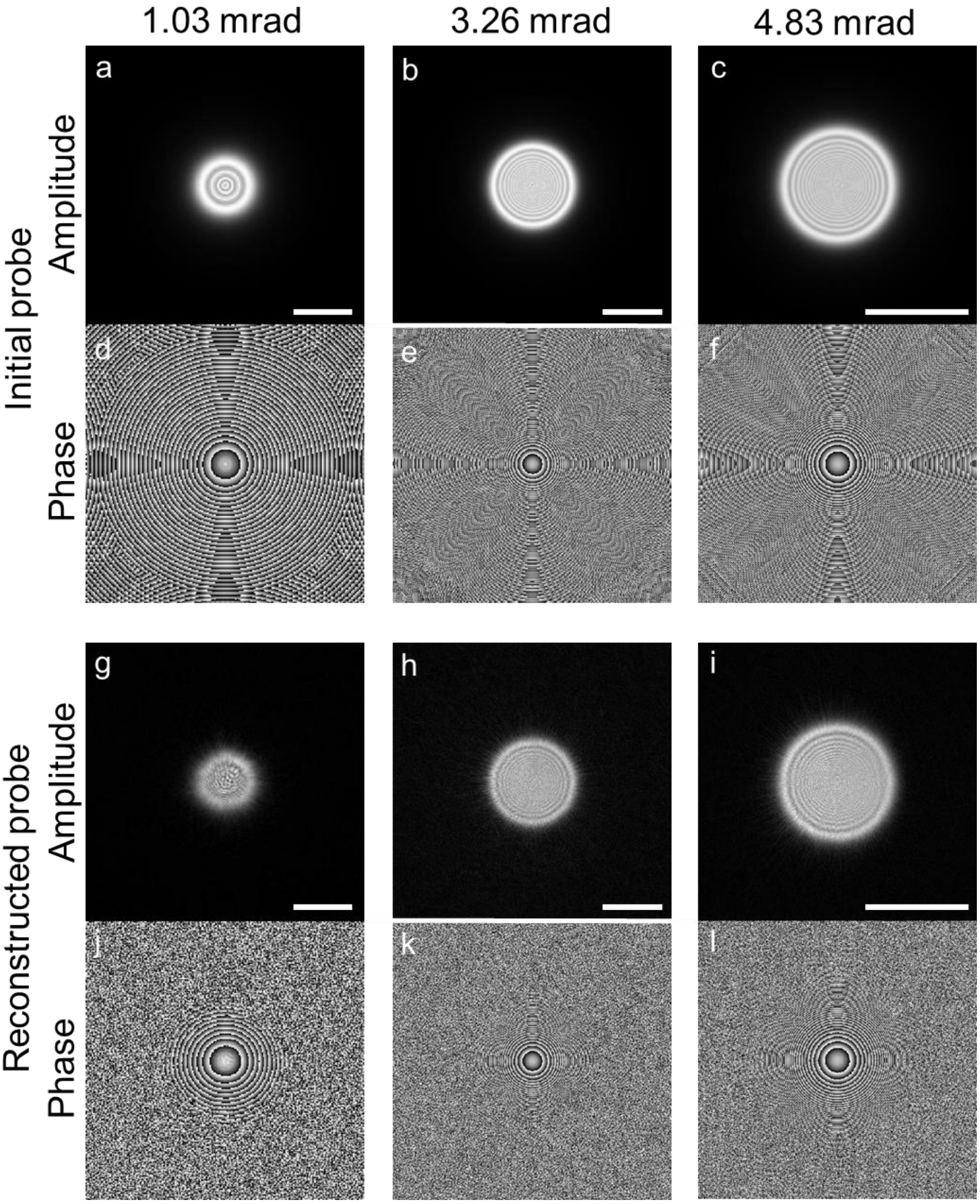

Supplementary Figure 10

Initial estimated and reconstructed probe functions for different CSA values. (**a** to **c**) Modulus and (**d** to **f**) phase of the initially estimated probe function. (**g** to **i)** Modulus and (**j to l)** phase of the probe function after reconstruction for α=1.03mrad (Left column), 3.26mrad (Middle column) and 4.83mrad (Right column) corresponding to (**a** to **c**) and (**d** to **f**) selected from sets of typically 29, 22 and 22 reconstructed datasets. Scale bars: 25 nm.

Rotavirus

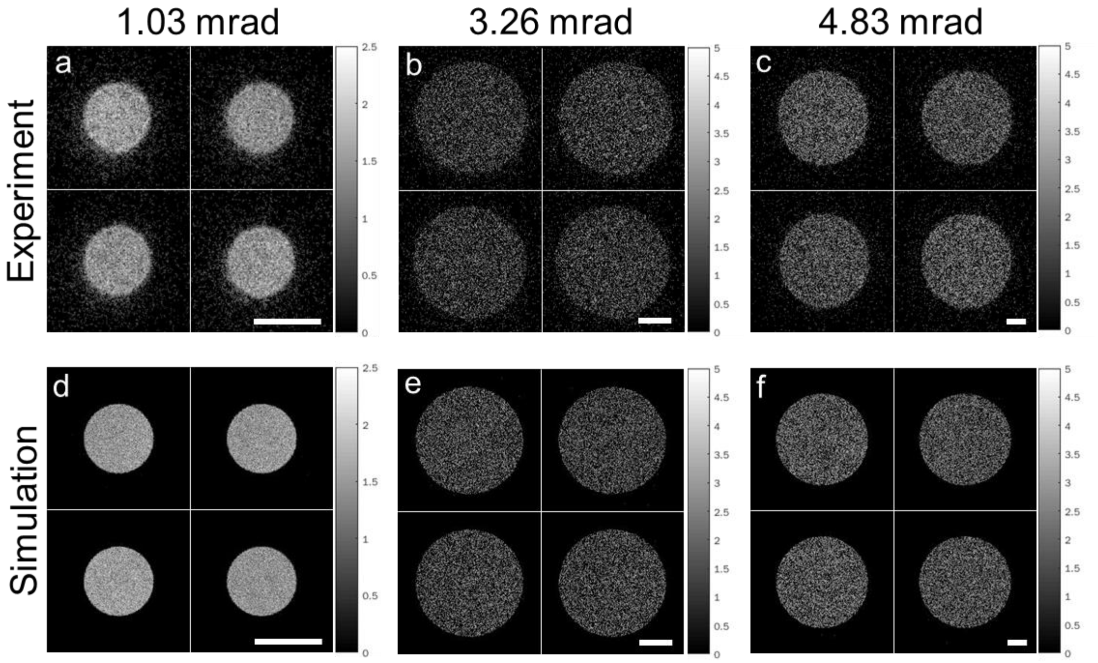

Apoferritin

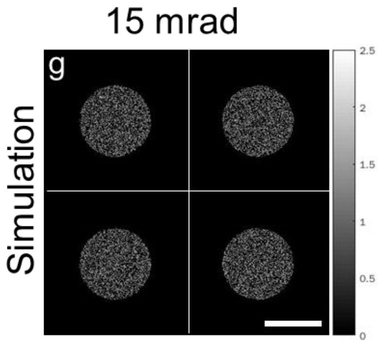

Supplementary Figure 11

Typical 2 × 2 subsets of (**a** to **c**) experimental and (**d** to **f**) simulated diffraction patterns for α=1.03mrad (Left column), 3.26mrad (Middle column) and 4.83mrad (Right column). Scale bars: 2 mrad. (**g**) Typical 2 × 2 subsets of diffraction patterns for simulations of apoferritin corresponding to α=15 mrad. Scale bars: 12 mrad

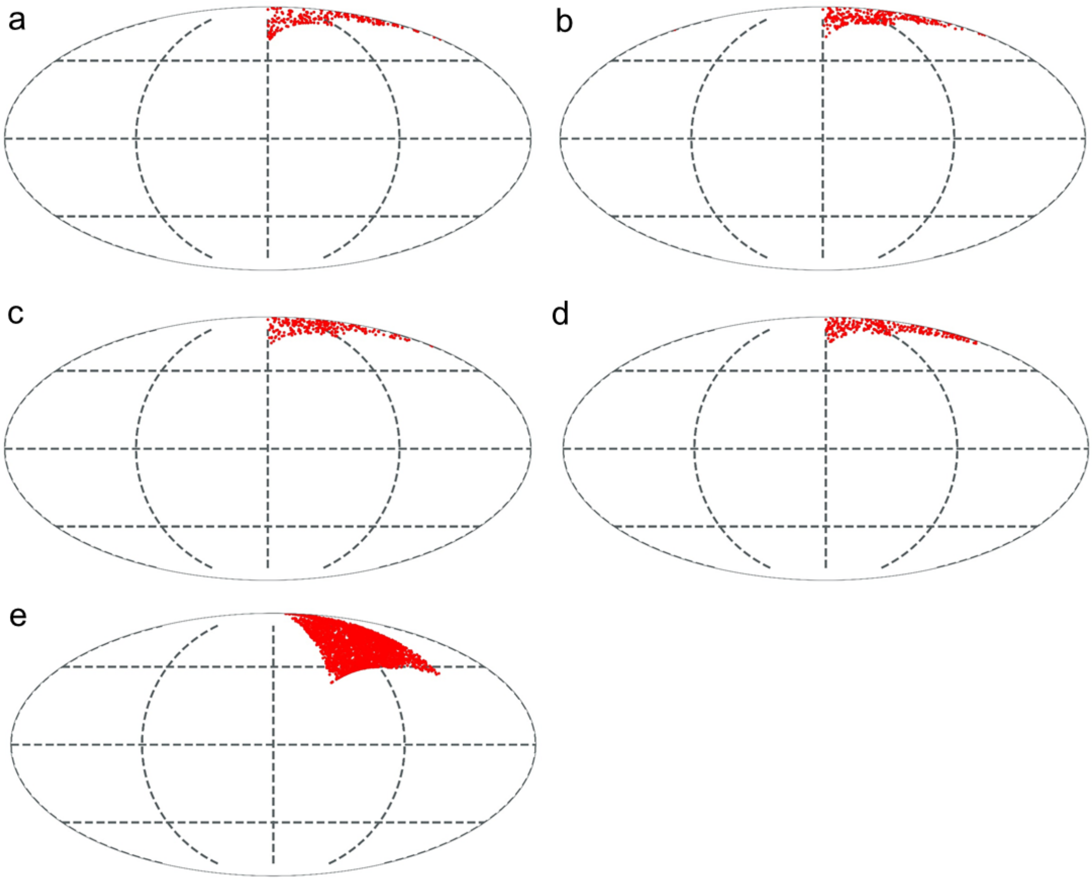

Supplementary Figure 12

Orientation distributions[2] of particles used for SPA.

(**a to c**) Orientation distributions corresponding to rotavirus DLPs for α=1.03mrad (232 particles), 3.26 mrad (318 particles) and 4.83mrad (241 particles). (**d**) Orientation distribution corresponding to rotavirus DLPs of the test subsets (269 particles) from cryo-TEM data. (**e**) Orientation distribution of apoferritin particles in the ptychpgraphic SPA simulation for α=15 mrad (2826 particles).

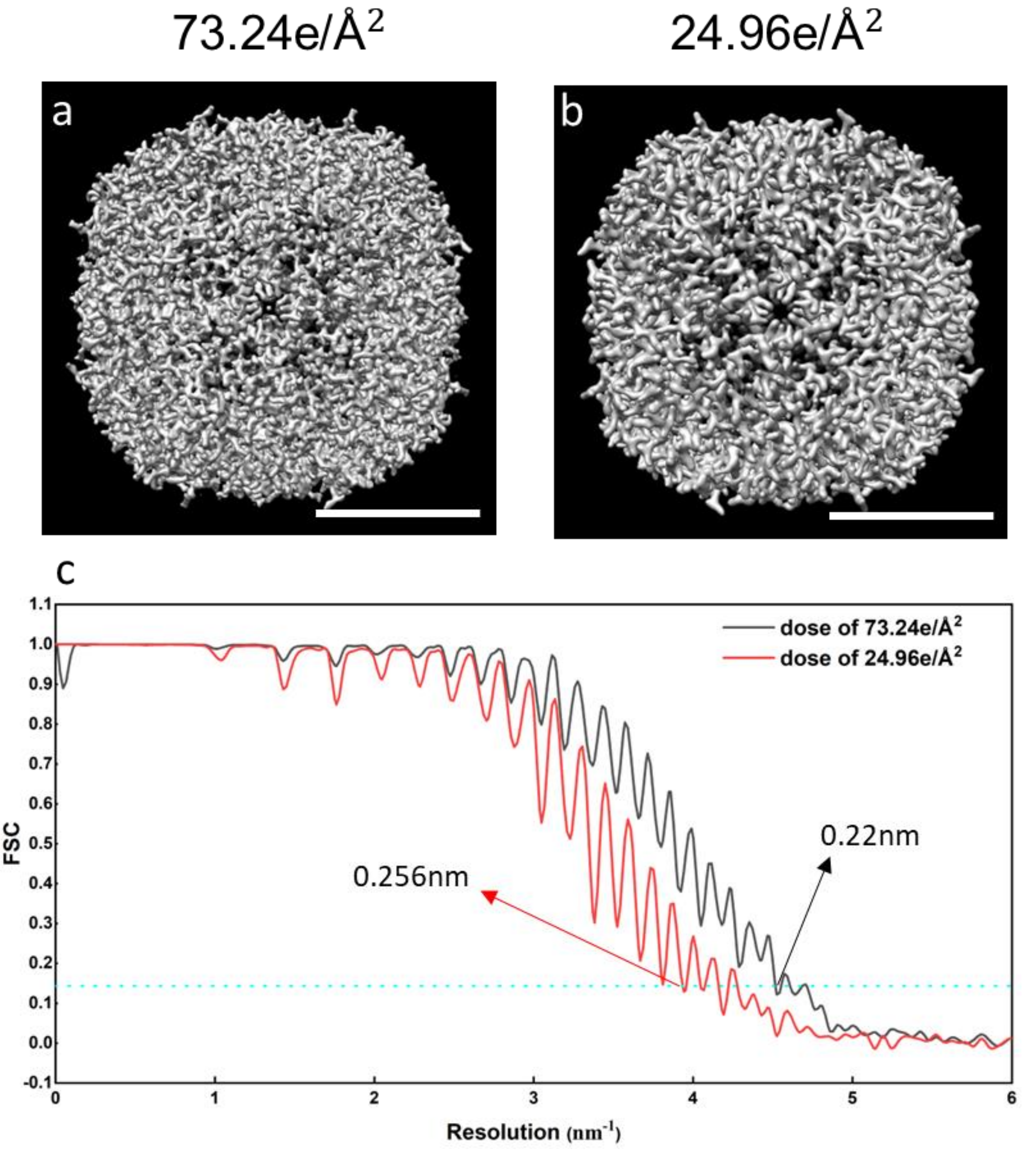

Supplementary Figure 13

Dose-dependent EPty-SPA simulations of apoferritin at atomic resolution. (**a** and **b**) Simulations of the ptychographic 3D map at dose of 73.24 e/Å^2 and 24.96 e/Å^2. (**c**) Fourier shell correlation curves of the 3D maps in (**a**) and (**b**). Scale bars: 5 nm (**a** and **b**)

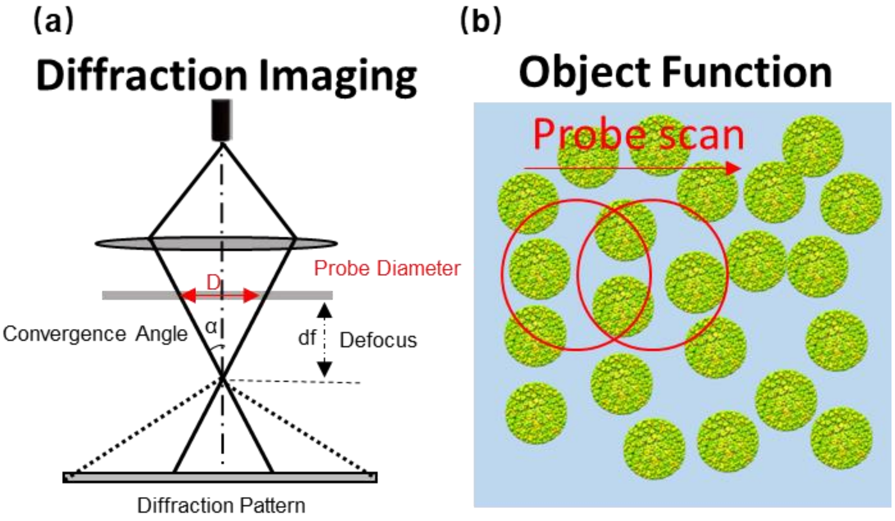

Supplementary Figure 14
(**a**) Schematic diagram of the optical configuration used for ptychography. (**b**) Overlapping region between adjacent probe positions on the sample plane.

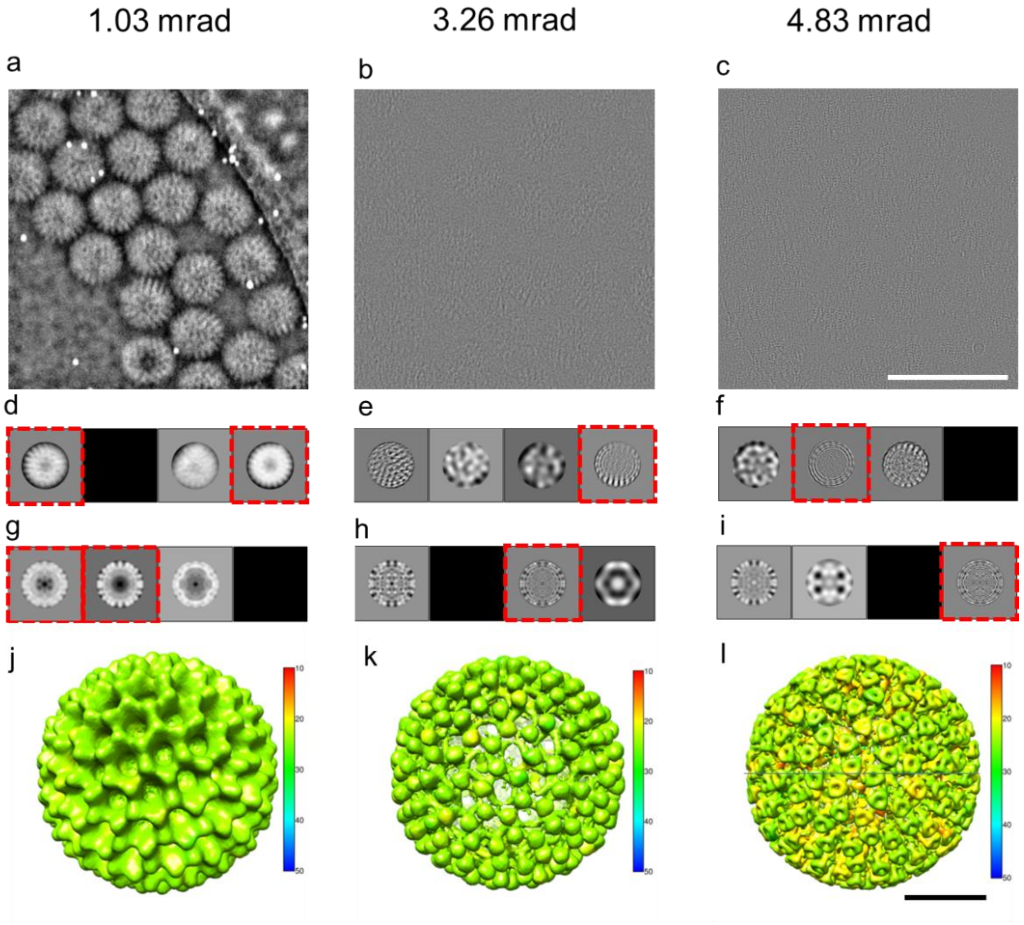

Supplementary Figure 15

Cryo Ptychographic SPA data processing workflow at various CSAs (Left column: 1.03 mrad; Middle column: 3.26 mrad; Right column: 4.83 mrad;). (**a** to **c**) Representative 2D ptychographic phase images, (**d** to **f**) 2D-classes (classes selected in red box are used to perform 3D classification),(**g** to **i**) central slices of 3D-classes (classes selected in red box are used to perform final 3D Refine),(**j** to **l**) the maps colored to resolution with a local resolution estimate at range of 10 Å to 50 Å) . (the details of parameters used in each step of workflow are given in Supplementary Table 2). Scale bars: 160 nm (**a** to **c**), 25 nm (**j** to **l**).

Supplementary Table 1. Parameters for cryo-ptychographic experiments

Dataset	Setting (1)	Setting (2)	Setting (3)
Sample	Rotavirus	Rotavirus	Rotavirus
Accelerating voltage (**kV**)	300	300	300
Convergence semi-angle (**mrad**)	1.03	3.26	4.83
Maximum detection semi-angle (**mrad**)	2.12	4.34	7.51
Defocus (**µm**)	-13.5	-4.5	-3
Probe size (**nm**)	27.92	29.34	28.96
Diffraction pattern dimension (**pixels**)	256×256	515×515	515×515
Step size (**nm**)	3.125	3.117	3.086
Raster scan dimension	127×127	255×255	255×255
Beam current (p**A**)	4	4	4
Dwell time per diffraction pattern (**ms**)	1	1	1
Counts per diffraction pattern	86612	84642	92581
Overlap ratio	0.858	0.865	0.865
Dose ($\mathbf{e}/\mathbf{Å}^2$)	22.64	24.07	24.57
Redundancy $\boldsymbol{\sigma}_{\mathbf{pty}}$	217.13	335.83	154.96

Supplementary Table 2. Parameters used for single-particle reconstructions

Dataset	Setting (1)	Setting (2)	Setting (3)	Setting (4)
Sample	Rotavirus	Rotavirus	Rotavirus	Rotavirus
Accelerating voltage (**kV**)	300	300	300	300
Convergence semi-angle (**mrad**)	1.03	3.26	4.83	N/A (TEM)
Initial pixel size (**nm**)	0.465	0.227	0.131	0.1205
Initial box size	248	508	880	956
Rescaled pixel size (**nm**)	0.465	0.465	0.465	0.465
Rescaled box size (**pixels**)	248	248	248	248
Picked particle quantity	257	443	498	378
After 2D classification	240	336	286	290
After 3D classification	232	318	241	269
Fourier shell correlation resolution (**nm**)	3.72	3.29	1.86	2.09

Supplementary Table 3. Parameters for cryo-ptychographic simulations

Dataset	Setting (1)	Setting (2)	Setting (3)	Setting (4)
Sample	Rotavirus	Rotavirus	Rotavirus	Apoferritin
Accelerating voltage ($\mathbf{kV}$)	300	300	300	300
Convergence semi-angle ($\mathbf{mrad}$)	1.03	3.26	4.83	15
Maximum detection semi-angle ($\mathbf{mrad}$)	2.12	4.34	7.51	30
Defocus ($\mathbf{\mu m}$)	-13.5	-4.5	-3	-0.5
Probe size ($\mathbf{nm}$)	27.92	29.34	28.96	15
Diffraction pattern dimension ($\mathbf{pixels}$)	256×256	515×515	515×515	800×800
Step size ($\mathbf{nm}$)	3.255	3.178	3.144	1.509
Raster scan dimension	255×255	109×109	60×60	40×40
Counts per diffraction pattern	88000	88000	88000	88000
Overlap ratio	0.852	0.862	0.862	0.872
Dose ($\mathbf{e/Å^2}$)	22.22	21.37	19.54	73.24
Redundancy $\boldsymbol{\sigma}_{\mathbf{pty}}$	461.25	327.81	108.74	64.65

Supplementary Table 4. Resolution of ptychographic SPA 3D maps as a function of motion factor for different CSAs

Motion Factor (**nm**)	Resolution (**nm**)		
	1.03 mrad	3.26 mrad	4.83 mrad
0	1.58	0.77	0.50
0.1	1.92	0.99	0.70
0.2	2.68	1.52	0.92
0.3	3.12	2.10	1.24
0.4	3.60	2.31	1.70
0.5	4.44	3.04	1.83

Supplementary Table 5. Selected information transfer bandwidths for different CSAs

Convergent semi angle (**mrad**)	Selected bandwidth (nm^{-1})
1.03	0 – 0.1691
3.26	0.1691 – 0.2298
4.83	0.2298 – 0.76

Supplementary Table 6. Normalized cross-correlation values between the experimental and simulated maps as a function of the motion factor for different CSAs

Motion factor (**nm**)	Normalized cross-correlation value		
	1.03 mrad	3.26 mrad	4.83 mrad
0	0.812	0.876	0.765
0.1	0.861	0.918	0.874
0.2	0.928	0.966	0.972
0.3	0.956	0.987	1
0.4	0.983	0.996	0.989
0.5	0.998	1	0.967
0.6	1	0.998	-
0.7	0.968	0.990	-

Supplementary Notes

Supplementary Note 1: Experimental configurations

TEM sample preparation of rotavirus double layered particles (DLPs) for cryo-ptychography

A suspension of Rotavirus DLPs (strain SA11) was prepared using a previously described method[3]. A suspension of DLPs was diluted in 10mM Tris HCl (pH 8.0) together with colloidal AuNPs ~ 6 nm in diameter at a concentration of 8 mg/ml. Four μl of the DLPs/AuNPs suspension was dropped onto holey carbon EM grids (Quantifoil) that had been plasma-cleaned for 30 s. Each grid was blotted for 5 s and subsequently plunged into a liquid ethane/propane mixture cooled by liquid nitrogen using a Gatan CP3 semi-manual plunger at 80 % humidity. The grids were then transferred and stored under liquid nitrogen.

Cryo-ptychography experiments of rotavirus DLPs

Cryo-ptychography experiments were performed in scanning diffraction mode on a JEOL ARM 300CF operated at 300kV with data recorded on a pixelated detector (Merlin 4R[4], 512 x 512 pixels) The vitrified EM samples were transferred into a Gatan 698 Elsa cryo-transfer holder to maintain the specimen temperature at -176 ± 2 °C. Three values of the CSA of 1.03 mrad, 3.26 mrad and 4.83 mrad were used for ptychographic 4D datasets. Detailed experimental parameters for each convergence semi-angle are given in Supplementary Table 1. A convergence semi-angle of 4.83 mrad is used here as an example to illustrate the relevant parameters used. A probe with a CSA of 4.83 mrad and a defocus of -3 μm (underfocus) gives a probe diameter of 28.96 nm at the sample (Supplementary Fig. 10c). This was raster scanned over the sample area in an array of 255 × 255 probe positions with a step size of 3.1 nm giving an overlap ratio of 86.5%. At each probe position, a diffraction pattern was recorded on a pixelated detector with an effective pixel size of 0.029 mrad and a dwell time of 1ms. Typical 2 × 2 subsets of diffraction patterns for the experiments are given in Supplementary Fig. 11 a-c. The beam current was 4 pA for the three vales of α used, corresponding to an electron dose of 24.57 e/Å^2. Details of the dose calculations are given in Ref[5].

Supplementary Note 2: Ptychographic reconstruction configurations

Ptychographic reconstructions were carried out using the ePIE[6] algorithm. Both probe and object functions were reconstructed after 100 iterations. The estimated and reconstructed probe functions using the three CSAs given in Supplementary Table 1 are shown in Supplementary Fig. 10. The overlap ratio of all the ptychographic 4D datasets was around 86% and the degree of redundancy was estimated as 217, 336 and 155 for datasets with CSAs of 1.03, 3.26 and 4.83 mrad. Details of the redundancy calculation are provided in Supplementary Note 6.

Supplementary Note 3: Single particle reconstruction

Typical ptychographic reconstructions of rotavirus DLPs in vitrified ice at different CSA values (Left column: 1.03 mrad; Middle column: 3.26 mrad; Right column: 4.83 mrad;) are shown in Supplementary Fig. 1. Using the e2box.py program in EMAN2.2[7], 257, 443, 498 and 378 particles (a typical single particle is shown in Fig. 3a-c,e) were picked for CSA values of of 1.03 mrad, 3.26 mrad, 4.83 mrad and TEM. Subsequently, 2D classification was performed using the software package Relion 3.1[8] followed by initial model calculation for 300 iterations with I2 symmetry and an angular sampling of 3.7 degree based on the SGD[9] (stochastic gradient descent) algorithm. The 3D classification was performed for 40 iterations with a regularisation parameter of 4. A final selection of particles from the 3D classification was carried out and these particles were used for the final 3D refinement using maximum a posteriori (MAP)[10]. The optimal model generated from the 3D classification was used as the initial reference map for 3D refinement until convergence[10] using a low-pass filter at 5 nm with I2 symmetry imposed. The convergence criterion is that no resolution improvement or orientation and translation changes occurs for at least two iterations[10].

Finally, the orientation distribution[2] of the particles used for map optimisation was measured as shown in Supplementary Fig.12. The resolution was given by Fourier shell correlation[11] as shown in Fig. 4 (main Text).

Supplementary Note 4: Ptychographic simulations of Rotavirus DLPs and Apoferritin

To validate our experimental results, multislice simulations of ptychographic SPA using a model of rotavirus DLPs and the same parameters as the experiments were calculated. To explore the full potential for atomic resolution cryo-EPty SPA, a simulation with α=15 mrad was also calculated for a model of apoferritin (PDB-7A6A) without beam-induced motion. The parameters of the experiments and simulations are listed in Supplementary Table 1 and 3, respectively. The atomic potential map was built from the 3KZ4 (Rotavirus) and 7A6A (apoferritin) models taken from the protein data bank (PDB) and calculations used the InSiliconTEM[12] Matlab code. Sample thicknesses of 71 nm for rotavirus and 15 nm for apoferritin were used. The isolated atom superposition approximation (IASA) was used because the solvent has a negligible effect on the contrast of the sample under low electron dose[13] conditions.

The interaction complex potential field of a whole macromolecule embedded in ice can hence be written as[12]:

$$V_{total} = V_{ph} + i * V_{ab} \quad (1)$$

where V_{ph} is the real part of the total interaction potential calculated by the IASA to model the interaction between the incident electron wave and a macromolecule embedded in ice; V_{ab} is the imaginary part of the total

interaction to model the effects of inelastic scattering described by the inelastic mean free path, Λ_{in} and can be written as:

$$V_{ab} = \frac{1}{2\sigma\Lambda_{in}} \qquad (2)$$

where $\sigma = \frac{2m|e|\lambda}{h^2}$ is the interaction constant, and λ, e, and m are the relativistic wavelength, charge and mass of the incident electrons, respectively.

A whole molecuar motion induced by the beam is recognised as one of the main factors attenuating high-resolution information in cryo-EM[14-16]. This motion of the specimen during acquisition leads to significant blurring in the images, which causes the experimental contrast to be significantly lower than theoretical predictions for conventional phase contrast TEM imaging, particularly at high spatial frequencies. The effects of the beam-induced movement of the whole molecular on the contrast can be modelled empirically as[12]:

$$V_{ph}^{'}(\vec{\mathbf{q}}) = V_{ph}(\vec{\mathbf{q}})\exp(-2\pi^2\sigma_m^2|\vec{\mathbf{q}}|^2) \qquad (3)$$

where $\vec{\mathbf{q}}$ is a 3D vector in Fourier space and σ_m is an isotropic motion factor, which blurs the interaction potential; $V_{total}(\vec{\mathbf{q}})$ and $V_{total}^{'}(\vec{\mathbf{q}})$ are Fourier transforms of the original interaction potential with V_{total} given by Equation (1) and resultant interaction potential $V_{total}^{'}$, respectively.

In the simulations used in this work, we adapted this method by using a motion factor σ_m of 0.47±0.13 nm [17] which gives the best fit to the effects of beam-induced motion observed in our experimental data as shown in Supplementary Fig. 5.

Using this interaction potential, the propagation of a scanning convergent beam through multiple slices of the 3D potential as a function of probe position[12,13] was simulated. At each position, the intensity that best matched the experimental diffraction patterns was calculated with added Poisson noise corresponding to a total incident electron counts of $8.8e^4$ electrons added to model the detector noise according to[13]

$$I = Poisson(|ft(\psi_{exit})|^2) \qquad (4)$$

where ft and ft^{-1} denote a forward and inverse Fourier transform, respectively;

In equation 4 $Poisson(\mu)$ denotes the Poisson noise yield and returns a random number from a Poisson distribution with an expectation value, μ. Detailed simulation parameters for each CSA are given in Supplementary Table 3 and typical 2×2 diffraction pattern subsets of rotavirus DLPs and apoferritin for the simulations are shown in Supplementary Fig. 11 d-f and g, respectively.

Supplementary Note 5: Evaluation of the degree of fitting and motion blur

The degree of fitting between the experimental and simulated maps were performed using "*fit in map*" in the CHIMERA[1] software.Two quantities $ovelap$ and $correlation$ are defined as:

$$ovelap = <\vec{\mathbf{u}}, \vec{\mathbf{v}}> \quad (5)$$

$$correlation = \frac{ovelap}{|\vec{\mathbf{u}}||\vec{\mathbf{v}}|} \quad (6)$$

The $ovelap$ can be expressed as the inner product of the vectors $\vec{\mathbf{u}}$ and $\vec{\mathbf{v}}$ containing the experimental map values and the corresponding simulated map values. The calculation can include all non zero-valued experimental map grid points or only those with values above a map lowest contour level. The $correlation$ serves as a measure of the similarity between the experimental and simulated maps at different motion blurs, as shown in Supplementary Table 6 and plotted in Supplementary Fig. 5.

Here, it is assumed that the motion factor is an intrinsic whole-molecure motion induced by the beam that exists in the ptychographic experiment and it is mainly dependent on the size and dose rate of the incident beam given that the samples and microscopes are same. Since the probe properties (i.e. size, dose rate, exposure time and scanning step) at the sample plane were setup to be same for all three angles, the motion factor is considered as being independent of the angles. Therefore, the intrinsic motion factor for the current experimental system was evaluated by normalising cross-correlation values for each value of α between the experimental and simulated maps, and calculating the average and standard deviation of the data points for normalised cross-correlation values greater than 98% for all three angles. This gives an averaged motion factor of 0.47 ± 0.13 nm for the current experimental system.

Supplementary Note 6: Evaluation of the degree of redundancy of a dataset

A quantity σ_{pty} can be defined to estimate the degree of redundancy[18] of a ptychographic dataset as:

$$\sigma_{pty} = \frac{N * Q}{2 * (Q + O)} \quad (7)$$

where *N* represents the number of diffraction patterns used for reconstruction, *Q* is the total number of pixels in each diffraction pattern and *O* is the total number of pixels in the reconstructed object. Using the dataset for Setting (3) in Supplementary Table 1 as an example, the reconstructed object consisted of 7442 × 7442 pixels. In this case only the central 515 × 515 pixels in the diffraction patterns were used. The number of recorded diffraction patterns is 255 × 255 and therefore, the total number of pixels in the diffraction patterns used for reconstruction, (J) is $255^2 \times 515^2$, while the number of unknown pixels in the specimen and probe is $2 \times (7442 \times 7442 + 515^2)$. Hence, σ_{pty} is equal to

155, implying that the data collection settings are suitable for ptychographic reconstruction[18] in this dataset.

Supplementary Note 7: Time cost for ptychographic data collection and processing

For the experiment at Setting 1 in Supplementary Table 1 as an example, requires approximately 1 min to acquire one ptychographic dataset of 127 × 127 diffraction patterns using a 256 × 256 pixel detector with a frame time of 1ms. For processing, approximately 400 s is required to reconstruct a 2D phase from this dataset after 50 iterations of ePIE. Recently, detectors operating at 10,000 frames/s or faster have been developed or are under development[6]. In addition accelerated ptychographic reconstruction algorithms that can speed up the processing time by factors from 10 to 10^3 have been proposed for applications of real-time data processing and visualization (Real-time ptychography)[7].

Supplementary Note 8: Dose-dependent EPty-SPA simulations of apoferritin at atomic resolution

A range of the doses from 50e/ Å^2 for apoferritin[19] to 100 e/ Å^2 Rotavirus[20] have been used in the cryo-EM literature. Doses of 73.24 e/Å^2 and 24.96 e/Å^2 were simulated, and the results are shown as Supplementary Fig. 13 a) and b), respectively. The dose in the latter simulation (Supplementary Fig. 13b) was set to be similar to those for the Rotavirus experiments. Compared to the former with a higher dose, the attenable resolution is 0.256 nm, which is slightly worse than the value of 0.22 nm for a dose of 73.24 e/Å^2 as shown in Supplementary Fig. 13c.

Supplementary Note 9: Defocus settings in ptychography

The purpose of using a defocused probe in ptychography is different from that in TEM. In ptychography, the main purpose of using a defocused probe is to generate illumination (probe size in Supplementary Fig. 14 a) on the sample plane such that when scanning the probe on the sample, an overlap region is generated between adjacent probe positions (Supplementary Fig. 14 b). The probe diameter (D) is given geometrically as $D = 2(\alpha \times df)$. Therefore, for smaller CSA values, a larger defocus is needed for the same probe size. For $\alpha = 1.03$ mrad we used a defocus of $df = 13.5$ um, which gives a probe diameter of 27.92 nm. Using a step size of 3.125 nm, the overlap ratio between adjacent probe positions was estimated as 85.8%, larger than 60%, which implies that the ptychographic setup is suitably conditioned[21].

Supplementary Note 10: Cryo ptychographic SPA workflow

Supplementary Fig. 15 shows a typical cryo ptychographic SPA data processing workflow, which includes 2D-classes (Supplementary Fig. 15 d-f), 3D classes (Supplementary Fig. 15 g-i), the map colored to resolution, a local resolution estimate with FSC cut-off (Supplementary Fig. 15 j-l), as well as a

representation of the angular distribution (Supplementary Fig. 12 a-c) of particles used in the final reconstruction.The details of parameters used in each step of workflow are given in Supplementary Table 2.

Supplementary References

[1] Pettersen, E. F. *et al.* UCSF Chimera--a visualization system for exploratory research and analysis. *J. Comput. Chem.* **25**, 1605-1612 (2004).

[2] Naydenova, K. & Russo, C. J. Measuring the effects of particle orientation to improve the efficiency of electron cryomicroscopy. *Nat. Commun.* **8**, 629 (2017).

[3] Zhou, L. *et al.* Low-dose phase retrieval of biological specimens using cryo-electron ptychography. *Nat. Commun*. **11**, 2773 (2020).

[4] Mir, J. A. *et al.* Characterisation of the Medipix3 detector for 60 and 80keV electrons. *Ultramicroscopy* **182**, 44-53 (2017).

[5] Song, J. *et al.* Atomic Resolution Defocused Electron Ptychography at Low Dose with a Fast, Direct Electron Detector. *Sci. Rep.* **9**, 3919 (2019).

[6] Maiden, A. M. & Rodenburg, J. M. An improved ptychographical phase retrieval algorithm for diffractive imaging. *Ultramicroscopy* **109**, 1256-1262 (2009).

[7] Tang, G. *et al.* EMAN2: an extensible image processing suite for electron microscopy. *J. Struct. Biol.* **157**, 38-46 (2007).

[8] Scheres, S. H. RELION: implementation of a Bayesian approach to cryo-EM structure determination. *J. Struct. Biol*. **180**, 519-530 (2012).

[9] Bottou, L. *Large-Scale Machine Learning with Stochastic Gradient Descent*. (Physica-Verlag Gmbh & Co, 2010).

[10] Scheres, S. H. W. RELION: Implementation of a Bayesian approach to cryo-EM structure determination. *J. Struct. Biol.* **180**, 519-530 (2012).

[11] Penczek, P. A. in *Cryo-EM, Part B: 3-D Reconstruction Methods in Enzymology* 73-100 (2010).

[12] Vulovic, M. *et al.* Image formation modeling in cryo-electron microscopy. *J. Struct. Biol.* **183**, 19-32 (2013).

[13] Pelz, P. M., Qiu, W. X., Bucker, R., Kassier, G. & Miller, R. J. D. Low-dose cryo electron ptychography via non-convex Bayesian optimization. *Sci. Rep.* **7**, 9883 (2017).

[14] Glaeser, R. M. & Hall, R. J. Reaching the information limit in cryo-EM of biological macromolecules: experimental aspects. *Biophys. J.* **100**, 2331-2337 (2011).

[15] Henderson, R. Image contrast in high-resolution electron microscopy of biological macromolecules: TMV in ice. *Ultramicroscopy* **46**, 1-18 (1992).

[16] Brilot, A. F. *et al.* Beam-induced motion of vitrified specimen on holey carbon film. *J. Struct. Biol.* **177**, 630-637 (2012).

[17] Henderson, R. & Glaeser, R. M. Quantitative analysis of image contrast in electron micrographs of beam-sensitive crystals. *Ultramicroscopy* **16**, 139-150 (1985).

[18] Maiden, A. M., Humphry, M. J., Zhang, F. & Rodenburg, J. M. Superresolution imaging via ptychography. *J. Opt. Soc. Am. A* **28**, 604-612 (2011).

[19] Yip, K. M., Fischer, N., Paknia, E., Chari, A. & Stark, H. Atomic-resolution protein structure determination by cryo-EM. *Nature* **587**, 157-161 (2020).
[20] Grant, T. & Grigorieff, N. Measuring the optimal exposure for single particle cryo-EM using a 2.6 A reconstruction of rotavirus VP6. *Elife* **4**, e06980 (2015).
[21] Bunk, O. *et al.* Influence of the overlap parameter on the convergence of the ptychographical iterative engine. *Ultramicroscopy* **108**, 481-487 (2008).
[22] Philipp, H. T. *et al.* Very-High Dynamic Range, 10,000 Frames/Second Pixel Array Detector for Electron Microscopy. *Microsc. Microanal.*, 1-16 (2022).
[23] Marchesini, S. *et al.* SHARP: a distributed GPU-based ptychographic solver. *J Appl. Crystallogr.* **49**, 1245-1252 (2016).
