# Supplementary material for: Cryogenic electron ptychographic single particle analysis with wide bandwidth information transfer
Source: Nat Commun. 2023 May 25;14:3027. doi: 10.1038/s41467-023-38268-0 (PMC10212999; doi:10.1038/s41467-023-38268-0)
Supplement: Supplementary file 2 — Reporting Summary [file 41467_2023_38268_MOESM2_ESM.pdf]

nature portfolio

Corresponding author(s): Peng Wang, Angus I Kirkland

Last updated by author(s): Mar 17, 2023

Reporting Summary

Nature Portfolio wishes to improve the reproducibility of the work that we publish. This form provides structure for consistency and transparency in reporting. For further information on Nature Portfolio policies, see our Editorial Policies and the Editorial Policy Checklist.

Statistics

For all statistical analyses, confirm that the following items are present in the figure legend, table legend, main text, or Methods section.

n/a	Confirmed	
☐	☒	The exact sample size (*n*) for each experimental group/condition, given as a discrete number and unit of measurement
☐	☒	A statement on whether measurements were taken from distinct samples or whether the same sample was measured repeatedly
☒	☐	The statistical test(s) used AND whether they are one- or two-sided<br>*Only common tests should be described solely by name; describe more complex techniques in the Methods section.*
☒	☐	A description of all covariates tested
☒	☐	A description of any assumptions or corrections, such as tests of normality and adjustment for multiple comparisons
☐	☒	A full description of the statistical parameters including central tendency (e.g. means) or other basic estimates (e.g. regression coefficient) AND variation (e.g. standard deviation) or associated estimates of uncertainty (e.g. confidence intervals)
☒	☐	For null hypothesis testing, the test statistic (e.g. *F*, *t*, *r*) with confidence intervals, effect sizes, degrees of freedom and *P* value noted<br>*Give P values as exact values whenever suitable.*
☒	☐	For Bayesian analysis, information on the choice of priors and Markov chain Monte Carlo settings
☒	☐	For hierarchical and complex designs, identification of the appropriate level for tests and full reporting of outcomes
☒	☐	Estimates of effect sizes (e.g. Cohen's *d*, Pearson's *r*), indicating how they were calculated

Our web collection on statistics for biologists contains articles on many of the points above.

Software and code

Policy information about availability of computer code

Data collection	Electron microscopy data was collected on commercial softwares integrated to the transmission electron microscopy
Data analysis	The ePIE algorithm for data analysis has been described in published literatures, which are cited and described in the manuscript. The Relion3.1 and EMAN2.2 software for 3D reconstruction has been described in published literatures, which are cited in the manuscript

For manuscripts utilizing custom algorithms or software that are central to the research but not yet described in published literature, software must be made available to editors and reviewers. We strongly encourage code deposition in a community repository (e.g. GitHub). See the Nature Portfolio guidelines for submitting code & software for further information.

Data

Policy information about availability of data

All manuscripts must include a data availability statement. This statement should provide the following information, where applicable:

- Accession codes, unique identifiers, or web links for publicly available datasets
- A description of any restrictions on data availability
- For clinical datasets or third party data, please ensure that the statement adheres to our policy

The data that support the findings of this study are available from the corresponding author upon request

Human research participants

Policy information about studies involving human research participants and Sex and Gender in Research.

Reporting on sex and gender	Sex and gender are not involved in this research.
Population characteristics	No human participant is involved in this research.
Recruitment	No human participant is involved in this research
Ethics oversight	This is not necessary for this research

Note that full information on the approval of the study protocol must also be provided in the manuscript.

Field-specific reporting

Please select the one below that is the best fit for your research. If you are not sure, read the appropriate sections before making your selection.

☒ Life sciences ☐ Behavioural & social sciences ☐ Ecological, evolutionary & environmental sciences

For a reference copy of the document with all sections, see nature.com/documents/nr-reporting-summary-flat.pdf

Life sciences study design

All studies must disclose on these points even when the disclosure is negative.

Sample size	The sample size is calculated based on data redundancy. The redundancy = pixels per diffraction pattern / (2*pixels in specimen and probe reconstructions). The redundancy of the reconstruction dataset is larger than 1, which is sufficient for the reconstruction algorithm. Rotavirus double layered particles are used as sample. A total of 3 different data sets were acquired at convergence semi-angles (CSA) of 1.03, 3.26 and 4.83 mrad with a total of 49, 22 and 22 micrographs.
Data exclusions	All diffraction patterns that were collected were included in the analysis. There is no data exclusion.
Replication	Two independent maps were refined for rotavirus in order to estimate resolution according to the recommended procedures in the field (the 'gold standard')
Randomization	The samples were randomly distributed onto the electron microscopy grids;Division of datasets into two random halves was done based on standard approach in RELION 3.1.
Blinding	Blinding was not applicable to this study because this type of study does not use group allocation.

Reporting for specific materials, systems and methods

We require information from authors about some types of materials, experimental systems and methods used in many studies. Here, indicate whether each material, system or method listed is relevant to your study. If you are not sure if a list item applies to your research, read the appropriate section before selecting a response.

Materials & experimental systems

n/a	Involved in the study	
☒	☐	Antibodies
☒	☐	Eukaryotic cell lines
☒	☐	Palaeontology and archaeology
☒	☐	Animals and other organisms
☒	☐	Clinical data
☒	☐	Dual use research of concern

Methods

n/a	Involved in the study	
☒	☐	ChIP-seq
☒	☐	Flow cytometry
☒	☐	MRI-based neuroimaging
